# MSCs mediate long-term efficacy in a Crohn’s disease model by sustained anti-inflammatory macrophage programming via efferocytosis

**DOI:** 10.1038/s41536-024-00347-1

**Published:** 2024-01-20

**Authors:** Maneesh Dave, Atul Dev, Rodrigo A. Somoza, Nan Zhao, Satish Viswanath, Pooja Rani Mina, Prathyush Chirra, Verena Carola Obmann, Ganapati H. Mahabeleshwar, Paola Menghini, Blythe Durbin-Johnson, Jan Nolta, Christopher Soto, Abdullah Osme, Lam T. Khuat, William J. Murphy, Arnold I. Caplan, Fabio Cominelli

**Affiliations:** 1 Division of Gastroenterology and Hepatology, Department of Internal Medicine, UC Davis Medical Center, University of California Davis School of Medicine, Sacramento, CA USA; 2 Institute for Regenerative Cures, University of California Davis School of Medicine, Sacramento, CA USA; 3 Skeletal Research Center, Department of Biology, Case Western Reserve University, Cleveland, OH USA; 4 Division of Gastroenterology and Liver Disease, University Hospitals, Case Western Reserve University, Cleveland, OH USA; 5 Department of Biomedical Engineering, Case Western Reserve University, Cleveland, OH USA; 6 Department of Diagnostic, Interventional and Pediatric Radiology, Inselspital, Bern University Hospital, University of Bern, Bern, Switzerland; 7 Department of Pathology, School of Medicine, Case Western Reserve University, Cleveland, OH USA; 8 Division of Biostatistics, Department of Public Health Sciences, University of California Davis School of Medicine, Sacramento, CA USA; 9 Division of Malignant Hematology/Cell and Marrow Transplantation, Department of Internal Medicine, University of California Davis School of Medicine, Sacramento, USA; 10 Department of Dermatology, University of California Davis School of Medicine, Sacramento, CA USA

**Keywords:** Crohn's disease, Crohn's disease

## Abstract

Mesenchymal stem cells (MSCs) are novel therapeutics for the treatment of Crohn’s disease. However, their mechanism of action is unclear, especially in disease-relevant chronic models of inflammation. Thus, we used SAMP-1/YitFc (SAMP), a chronic and spontaneous murine model of small intestinal inflammation, to study the therapeutic effects and mechanism of action of human bone marrow-derived MSCs (hMSC). hMSC dose-dependently inhibited naïve T lymphocyte proliferation via prostaglandin E_2_ (PGE_2_) secretion and reprogrammed macrophages to an anti-inflammatory phenotype. We found that the hMSCs promoted mucosal healing and immunologic response early after administration in SAMP when live hMSCs are present (until day 9) and resulted in a complete response characterized by mucosal, histological, immunologic, and radiological healing by day 28 when no live hMSCs are present. hMSCs mediate their effect via modulation of T cells and macrophages in the mesentery and mesenteric lymph nodes (mLN). Sc-RNAseq confirmed the anti-inflammatory phenotype of macrophages and identified macrophage efferocytosis of apoptotic hMSCs as a mechanism that explains their long-term efficacy. Taken together, our findings show that hMSCs result in healing and tissue regeneration in a chronic model of small intestinal inflammation and despite being short-lived, exert long-term effects via sustained anti-inflammatory programming of macrophages via efferocytosis.

## Introduction

Crohn’s disease (CD) affects more than 1 million individuals in the US and is becoming more common worldwide^1–3^. CD is thought to result from an inappropriate response of the host’s immune system to intestinal microbes^4^. Therefore, the existing management strategies target inflammation and include immunosuppressive therapy with corticosteroids, immunomodulators, monoclonal antibodies against cytokines, and anti-adhesion molecules^5^. However, only a fraction of patients who are started on these medications achieve and remain in remission for a long duration. In addition, the use of these immunosuppressive medications is associated with a risk of adverse events such as severe infections, and malignancies^6^. Thus, there is a need for novel therapies that induce sustained remission with minimal side effects to treat CD.

Cell-based therapies that utilize the immunosuppressive capacity of adult mesenchymal stem cells (MSCs) for immune-mediated diseases like CD are in clinical trials^7–11^. A phase III multicenter randomized placebo-controlled trial of allogeneic adipose-derived mesenchymal stem cells showed clinical and statistically significant healing of Crohn’s perianal fistulas and lead to its approval in European Union, Israel and Japan^12,13^. MSCs have good safety profile in clinical trials which has been attributed to their rapid clearance from the body after administration and yet have long term efficacy which is currently unexplained^14,15^. Furthermore, mechanistic studies in relevant and representative preclinical murine models of CD, especially chronic models are lacking. The studies performed to date, have demonstrated the benefit of MSC therapy in only acute models and target inflammation in the colon. The SAMP1/YitFc (SAMP) mice represent a unique model that spontaneously develops chronic small intestinal (SI) inflammation in the absence of genetic, chemical, and immunological manipulation^16^. This model also responds to conventional human CD therapies like anti-tumor necrosis factor-alpha agents^17^ and dexamethasone^18^. Thus, the SAMP mice, which closely mimic human CD, can be utilized as a high-fidelity preclinical model for CD therapies, therefore we investigated the efficacy and mechanism of human bone marrow-derived MSC (hMSC) therapy in SAMP mice.

## Results

### hMSCs suppress T cell proliferation in mixed lymphocyte reaction (MLR) and reprogram macrophages to an immunosuppressive phenotype

**Fig. 1:**
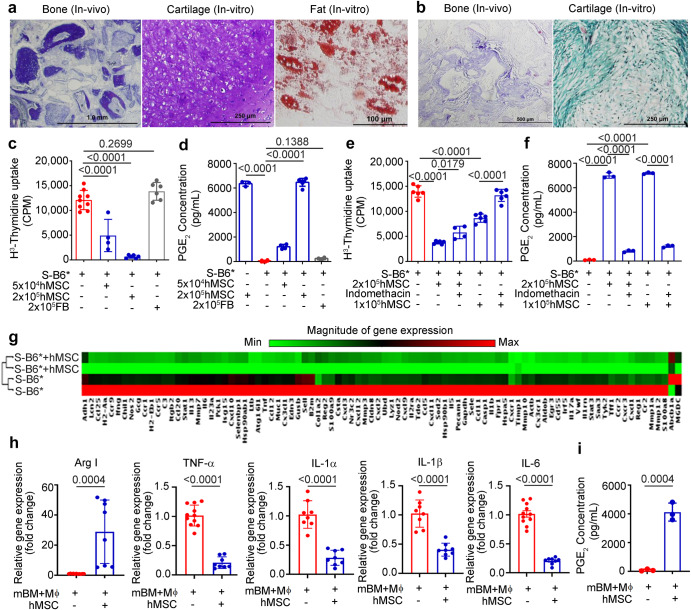
hMSC suppresses murine T cell proliferation and reprogram murine macrophages to an anti-inflammatory phenotype. **a** Mesodermal differentiation potential of BM-hMSCs and **b** Human fibroblasts (FB). **c** SAMP T cell proliferation in MLR (S: SAMP splenocytes, B6: C57Bl/6 J derived irradiated splenocytes) treated with hMSC and FB for 96 hours. **d** PGE_2_ concentration measured in mixed lymphocyte reaction (MLR) supernatants using an enzyme immunoassay. **e** SAMP T cell proliferation in MLR treated with hMSCs and COX inhibitor indomethacin (10 µM). **f** PGE_2_ concentration measured in MLR supernatant using an enzyme immunoassay. **g** Heat Map showing the gene expression of Crohn’s RT-PCR profiler array on MLR reaction, **h** Relative gene expression of indicated markers and cytokine measured in total RNA extracted from murine bone marrow-derived macrophages (mBM-Mϕ). The gene expression was determined by qRT-PCR, normalized to GAPDH, and expressed as fold change (2-^ΔΔCt^). **i** PGE_2_ concentration measured in reaction supernatant from hMSCs and murine bone marrow-derived macrophages (mBM-Mϕ) co-culture using an enzyme immunoassay. Data were expressed as mean ± SD from at least two independent experiments. *P* < 0.05 considered significant, by 1-way ANOVA (**c**–**f**) and 2-tailed, unpaired Student’s *t* test (**h, i**).

Human MSCs identified by expression of cell surface markers (CD73, CD90, CD105) and lack of hematopoietic marker CD34, CD14, and HLA-DR (Supplementary Fig. 1a) have progenitor cell capacities that can be activated during in-vitro cell culture to differentiate into multi lineages (Fig. 1a); human fibroblast served as study control and failed to differentiate (Fig. 1b). In addition to multilineage differentiation potential, MSCs have immunosuppressive properties^19^. We investigated the immunosuppressive potential of hMSCs in mixed lymphocyte reaction (MLR) and macrophage co-culture assay using immune cells from SAMP. In MLR, co-culturing of mixed cultures of lymphocytes (SAMP splenocytes stimulated by irradiated allogeneic, C57Bl/6 J derived splenocytes) with irradiated hMSC resulted in a dose-dependent inhibition of SAMP lymphocyte proliferation as measured by [3H] thymidine incorporation (*P* < 0.0001); human dermal fibroblasts served as cell therapy control and did not suppress lymphocyte proliferation (*P* = 0.2699) (Fig. 1c). A previous study has suggested that paracrine factors like prostaglandin E_2_ (PGE_2_) secreted by MSCs may be involved in therapeutic benefit^20^, therefore, we measured PGE_2_ secretion by hMSCs and in MLR supernatants using an enzyme immunoassay. hMSCs (2 × 10^5^) basally secreted PGE_2_ (6,355 ± 202.6 pg/mL), supernatants from MLR with hMSCs contained higher amounts of PGE_2_ (S-B6* + 2 × 10^5^ hMSCs: 6,464.04 ± 321.2 pg/mL) than proliferating lymphocytes (S-B6*: 49.64 ± 57.05 pg/mL; *P* < 0.0001) **(**Fig. 1d). To show that hMSCs suppress lymphocyte proliferation in MLR by prostaglandin E_2_ (PGE_2_) secretion, we blocked production of PGE_2_ in hMSCs using indomethacin, (pharmacological inhibitor of cyclooxegenase-1, cyclooxegenase-2 enzymes responsible for PGE_2_ production) in the MLR. We observed that indomethacin treatment rescued lymphocyte proliferation in MLR (*P* < 0.0001) (Fig. 1e) and confirmed a significant reduction in the PGE_2_ concentration in indomethacin treated samples [S-B6* + 2×10^5^hMSC:7000 ± 226.8 pg/mL vs S-B6* + 2 × 10^5 ^hMSC+Indo: 815.4 ± 83.94 pg/mL; *P* < 0.0001) (Fig. 1f). Furthermore, using the cells from MLR, we studied the expression of genes involved in Crohn’s disease pathogenesis using Crohn’s RT-PCR profiler array. hMSCs treatment resulted in the downregulation of 73 out of 84 pro-inflammatory CD genes (log2 fold change < 2; *Q* < 0.05) (Fig. 1g).

PGE_2_ has been shown to promote the polarization of macrophages to an anti-inflammatory phenotype^21^, therefore we next performed hMSCs co-cultures with bone marrow-derived macrophages from SAMP (mBM- Mϕ). In cell proximity co-culture, where hMSCs are in direct contact with macrophages, hMSCs educated mBM-Mϕ to an anti-inflammatory phenotype with increased gene expression of Arginase I (28.9-fold change, *P* = 0.0004), an anti-inflammatory phenotype marker and decreased gene expression of proinflammatory cytokines TNF-α (0.2-fold change, *P* < 0.0001), IL-6 (0.2-fold change, *P* < 0.0001), IL-1α (0.3-fold change, *P* < 0.0001), and IL-1β (0.4-fold change, *P* < 0.0001) (Fig. 1h). Like MLR, the supernatants from mBM-Mϕ + hMSC group had a significantly higher concentration of PGE_2_ (4120 ± 626.1 pg/mL; *P* = 0.0004) compared to mBM-Mϕ (Fig. 1i). Furthermore, in transwell co-culture assay the separation of hMSCs showed a similar trend with increased gene expression of Arginase-I (3.98-fold change, *P* < 0.0001) and decrease in gene expression of TNF-α (0.64-fold change, *P* < 0.001) (Supplementary Fig. 1b), however, we did not see significant changes in gene expression of IL-6, IL-1α, and IL-1β cytokines.

### Intraperitoneal hMSCs did not home to the small intestine in SAMP

**Fig. 2:**
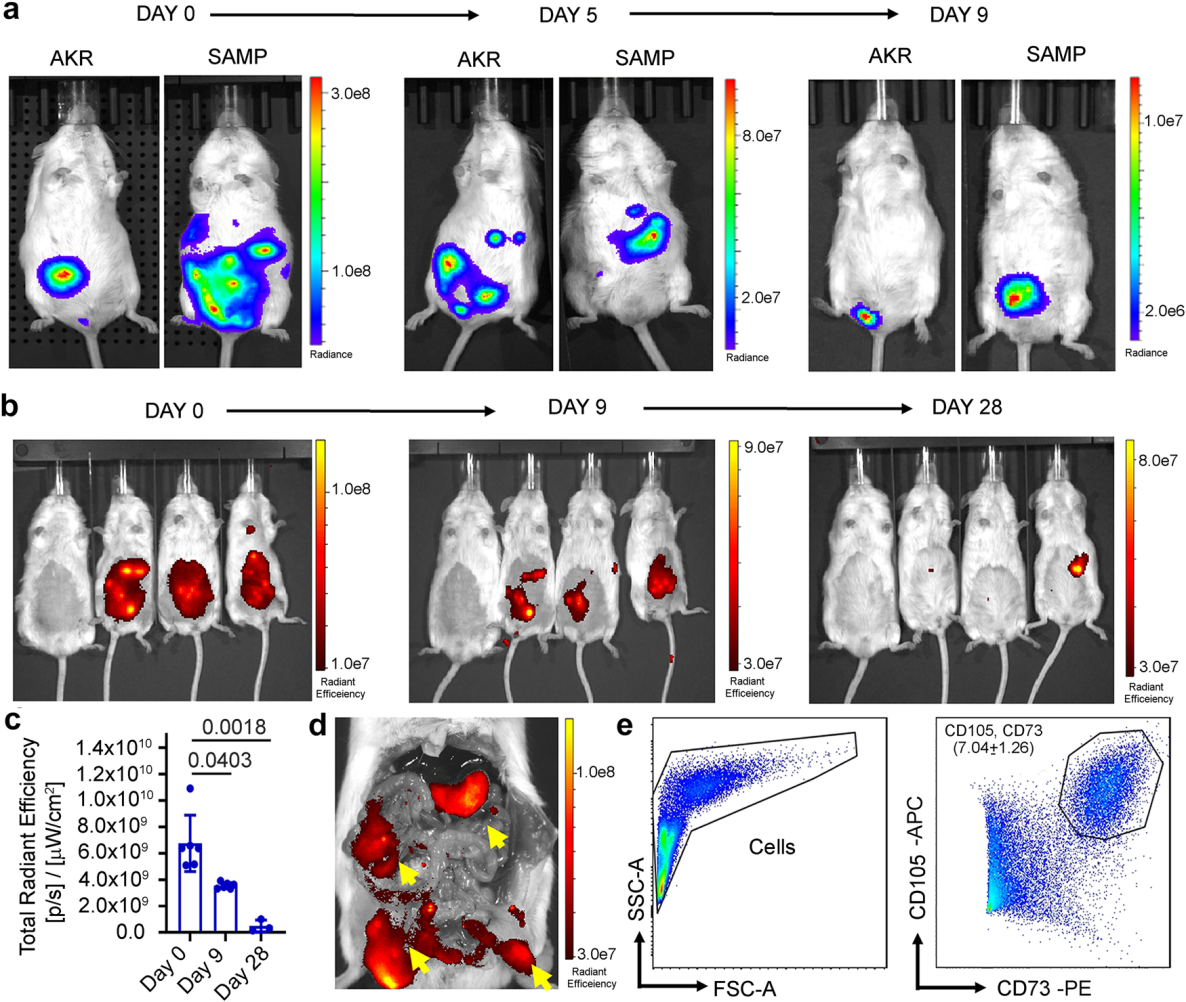
hMSC survive in the SAMP peritoneum cavity up to day 9. **a** Representative images showing time course BLI of transduced hMSCs in AKR and SAMP mice on days 0, 5, and 9. **b** Representative time course epifluorescence images of SAMP mice showing the presence of intraperitoneally administered NIR dye IVIS680 labeled hMSCs. Images were acquired on days 0, 9, and 28. **c** Quantitative estimation of total radiant efficiency in mice treated with IVIS680 tagged hMSCs. Data were expressed as mean ± SD from at least 2 independent experiments. *P* < 0.05 considered significant, by 1-way ANOVA. **d** Representative ex-vivo epifluorescence image of SAMP peritoneum cavity showing the presence of IVIS680 tagged hMSCs (yellow arrow) on day 9. **e** Flow cytometry gating scheme to detect hMSC cell population in the peritoneal lavage of hMSC administered SAMP at day 9.

Previous studies in the acute model of colitis have shown that intraperitoneal administration of MSCs can ameliorate colitis^22^, therefore we next studied the homing potential and survival of hMSC after intraperitoneal administration in SAMP mice. The hMSCs were transduced with lentivirus to express firefly luciferase and in vivo animal bioluminescent imaging (BLI) was performed to detect live hMSCs in diseased SAMP and non-disease control AKR mice. We observed a time course decline in the BLI signal intensity of bioluminescent hMSCs with a drop in signal intensity on day 5 followed by a weak detectable signal on day 9 (Fig. 2a); no signal was observed post-day 9. Ex vivo imaging performed on harvested body organs on day 9 showed a weak BLI signal (live hMSC) in the proximity of the stomach in SAMP with no evidence of hMSCs in the small intestine (Supplementary Fig. 2). To further confirm BLI data, we labeled hMSCs with near-infrared fluorescent surface dye and performed time course epifluorescence imaging in diseased SAMP (Fig. 2b). A similar trend of signal intensity was observed in epifluorescence imaging in SAMP, with a significant drop in signal intensity on day 9 (3.6e9 ± 0.23e9, *P* = 0.0403). Interestingly, we observed a very weak signal/hMSCs presence in the peritoneum cavity on day 28 (0.49e9 ± 0.44e9, *P* = 0.0018) (Fig. 2c) which is likely from the remnant of dead hMSCs as no live cells were observed by BLI or flow cytometry. We also performed ex vivo imaging of the peritoneum cavity in hMSC-administered SAMP and found a strong signal of IVISense680 tagged hMSC on day 9 (Fig. 2d) and a weak signal on day 28. We next performed flow cytometry on peritoneal lavage cells and demonstrated the presence of live hMSCs (CD105^+^, CD73^+^) on day 9 (Fig. 2e), however, no viable hMSCs were detected on day 28. Furthermore, flow cytometry performed on single cell suspension from the small intestine on day 9 and day 28 did not show the presence of hMSCs (Supplementary Fig. 3), confirming that the majority of hMSCs do not migrate to the small intestine.

### hMSCs result in mucosal healing and immunologic response early after administration

**Fig. 3:**
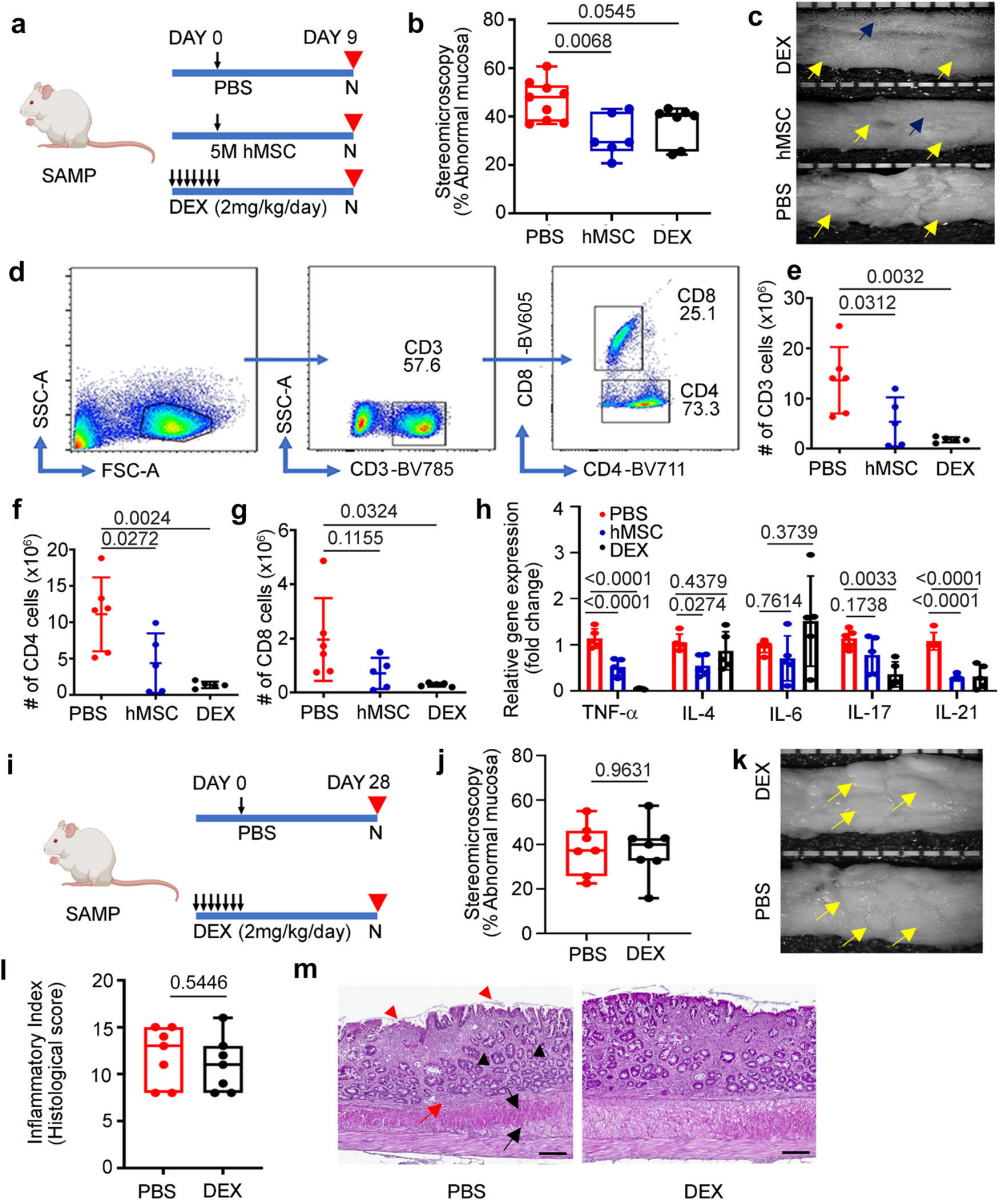
hMSC treatment shows mucosal healing and early sign of immunological response on day 9. **a** Schematic showing the treatment regimen of the experiment (*n* = 22). **b** Percent abnormal mucosa in PBS, hMSCs, and DEX-treated groups. **c** Representative comparative stereoscopic view of small intestine from DEX, hMSC, and PBS-treated groups (yellow arrow showing abnormal mucosa; blue arrow showing normal mucosa). **d** Flow cytometry gating strategy showing **e** Absolute number of CD3^+^, **f** CD4^+^, and **g** CD8^+^ cells in mesenteric lymph nodes (mLN). **h** Relative gene expression of cytokines from Th-1, Th-2, and Th-17 pathways was measured in total RNA extracted from mesenteric lymph node cells. Gene expression was determined by qRT-PCR, normalized to GAPDH, and expressed as fold change (2-ΔΔCt). **i** Schematic showing treatment regimen of the experiment (*n* = 14). **j** Percent abnormal mucosa in PBS and DEX-treated groups. **k** Representative comparative stereoscopic view of the small intestine in PBS and DEX treated groups (yellow arrows showing abnormal mucosa). **l** The inflammatory index for disease severity in SAMP mice treated with PBS and DEX. **m** Representative histopathology photomicrograph of ileum tissue from PBS, and DEX treated groups (Red triangle= villus distortion, black triangle=crypts hyperplasia, red arrow=immune infiltration in submucosa, black arrow= muscle hypertrophy). The scale bar represents 200 µm. Data were expressed as mean ± SD from at least two independent experiments unless specified. Each data point represents one mouse. In box and whiskers-plot, center line: median; box limits: 25-75 percentile; whiskers: min. to max. with all data points. *P* < 0.05, *P* < 0.01, *P* < 0.001 considered significant, by 1-way ANOVA and unpaired t-test (**j,** **l**).

As intraperitoneally injected live hMSC population were detected only till day 9, we studied their therapeutic response in SAMP mice with established chronic inflammation at day 9 after hMSC treatment. SAMP mice were treated with 5 million hMSCs; high-dose dexamethasone (DEX)^23^ served as the positive therapy control, while phosphate buffer saline (PBS) treated mice served as a negative control. Mice in the DEX group were injected with a daily dose of DEX for 7 days before the onset of the treatment regimen (Fig. 3a). 3D stereomicroscopy which quantifies spatial abnormalities (Supplementary Fig. 4) of the intestine^23^ showed mucosal healing with a significant decrease in the percent abnormal mucosa in the hMSC treatment group than PBS (PBS:51.08 ± 11.48 vs hMSC:25.78 ± 4.51; *P* = 0.0068) (Fig. 3b, c). Blinded histopathological scoring using a standardized, previously validated scoring system employed for SAMP mice^24^ showed a partial response in the DEX group (PBS:11.44 ± 2.65vs DEX:7.86 ± 2.12; *P* = 0.0563) while no response was observed in the hMSC group (PBS:11.44 ± 2.65 vs hMSC:12.33 ± 3.83; *P* = 0.8282) (Supplementary Fig. 5). As our in vitro data showed hMSCs induced T cell suppression, we studied the effect of hMSC treatment on the T cell population in mesenteric lymph node (mLN). We observed a significant decrease in absolute numbers of CD3^+^ (*P* = 0.0312) and CD4^+^ (*P* = 0.0272) cells; no significant decrease (*P* = 0.1155) was observed in CD8^+^ cells (Fig. 3d–g). Next, we studied gene expression of cytokines in mLN cells; hMSCs-treated mice showed a decrease in gene expression of cytokines involved in the Th-1 pathway (TNF-α; 0.52-fold change, *P* < 0.0001); Th-2 pathway (IL-4; 0.54-fold change, *P* = 0.0274), and Th-17 pathway (IL-21; 0.28-fold change, *P* < 0.0001) (Fig. 3h).

We next investigated the long-term effects of DEX and hMSCs treatment in SAMP mice. As disease recurs after stopping steroids in CD patients, we first studied the long-term effects of DEX treatment in SAMP mice with established inflammation at day 28 (Fig. 3i). SAMP mice treated with DEX show a similar percentage of abnormal mucosa as the PBS-treated group (PBS:38.10 ± 11.34 vs DEX:37.79 ± 12.73; P = 0.9631) (Fig. 3j, k). Blinded histopathological scoring showed severe disease recurrence at day 28 with non-significant changes (PBS:12 ± 3.05 vs DEX:11 ± 2.94; *P* = 0.5446) in inflammatory index between PBS and DEX-treated SAMP (Fig. 3l, m).

### hMSCs alleviate established small intestinal inflammation in SAMP mice and result in mucosal, histological, and radiological healing by day 28

**Fig. 4:**
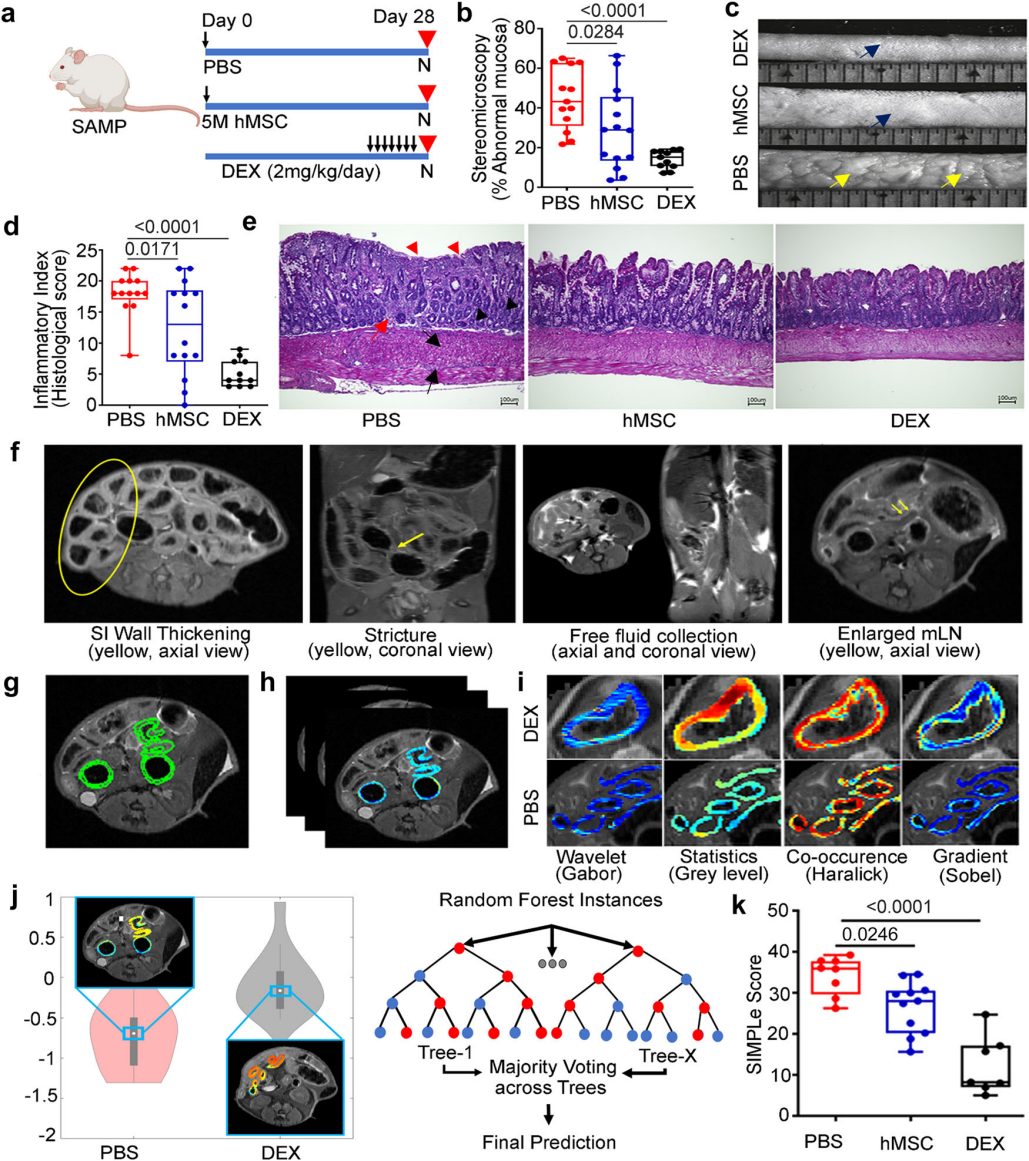
hMSC treatment alleviate intestinal inflammation in SAMP on day 28. **a** Schematic showing the treatment regimen of the experiment (n = 38). **b** Percent abnormal mucosa in PBS, hMSCs and DEX-treated groups. **c** Representative comparative stereoscopic view of the small intestine in DEX, hMSCs, and PBS-treated groups (yellow arrows showing abnormal mucosa; blue arrow showing normal mucosa). **d** The Inflammatory Index for disease severity in PBS, hMSCs, and DEX-treated groups showing histologic small intestine (SI) inflammation. **e** Representative histopathology photomicrograph of ileum tissue from PBS, hMSCs and DEX-treated groups (Red triangle = villus distortion, black triangle = crypts hyperplasia, red arrow = immune infiltration in submucosa, black arrow = muscle hypertrophy). The scale bar represents 200 µm. **f** MR image for SAMP shows human CD features, including SI wall thickening, stricture, free fluid collection, enlarged mesenteric lymph node(mLN). **g** MR image annotation in SAMP, **h** Radiomic feature extraction. **i** Radiomics analysis involved 100 radiomic features from four different classes to quantify SI wall appearance in terms of heterogeneity and gradient responses in PBS and DEX-treated groups. **j** Top-ranked features (Haralick and Sobel) were selected and used to train a machine-learning Random Forest classifier to yield a radiomics-based likelihood of disease severity. **k** SIMPle score resulted in further enhancement of disease severity assessment and discrimination between treatment groups (*n* = 26). Data were expressed as mean ± SD from at least two independent experiments unless specified, each data point represents one mouse. In the Box and whiskers-plot, center line: median; box limits: 25–75 percentile; whiskers: min. to max. with all data points. *P* < 0.05 considered significant, by 1-way ANOVA.

As there was an early but incomplete response after hMSC therapy, we next studied therapeutic response on day 28 of the treatment when no live hMSCs are present. SAMP mice treated with DEX show severe disease recurrence at day 28 after treatment, therefore, for the day 28 experiment, we modified our DEX treatment approach and administered it daily for 7 days just prior to euthanasia (Fig. 4a). In these experiments, SAMP mice were treated with 5 million hMSCs; no therapeutic response was observed with 2 million hMSCs (Supplementary Fig. 6). DEX served as the positive therapy control, while phosphate buffer saline (PBS) treated mice served as a negative control. hMSCs (PBS: 44.80 ± 15.57 vs. hMSC: 29.36 ± 20.29; *P* = 0.0284) and DEX (PBS: 44.80 ± 15.57 vs. DEX: 14.17 ± 4.56; P < 0.0001) treated mice had mucosal healing as demonstrated by a significant reduction in percent abnormal mucosa (Fig. 4b, c). The Inflammatory Index for disease severity is a histological index composed of the villus distortion index, active inflammation index, mononuclear inflammation index, chronic inflammation index, and transmural inflammation index. SAMP mice treated with hMSCs had reduced severity of small intestine (SI) inflammation (PBS: 18 ± 3.55 vs. hMSC: 12.43 ± 7.57; P = 0.0171); SAMP mice treated with DEX (2 mg/kg/24 h i.p.) for 7 days prior to euthanasia had more reduction of SI inflammation as compared to the hMSC treatment, (PBS: 18 ± 3.55 vs. DEX: 5.18 ± 2.18; *P* < 0.001) (Fig. 4d, e) manifesting in more intact villi, reduced immune infiltrate and transmural inflammation. Next, we studied the gene expression of cytokines in mLN cells; hMSCs-treated mice showed a decrease in gene expression of pro-inflammatory cytokines from the Th-1 pathway (TNF-α and IFN-γ); Th-2 pathway (IL-6, IL-4) and Th-17 pathway (IL-17, IL-21). DEX served as a positive control for the treatment, and significantly downregulated gene expression of the majority of proinflammatory cytokines including TNF-α, IFN-γ, IL-6, IL-17, and IL-21 (Supplementary Fig. 7).

Radiologic healing in the small intestine (SI) Crohn’s disease is associated with a reduction in the risk of poor outcomes in CD patients^25^. We previously extracted radiomic features (quantitative computer-extracted image descriptions) from magnetic resonance imaging (MRI) of CD patients and showed their ability in combination with clinical phenotype to predict the risk of surgery^26^. Therefore, we studied radiologic healing in SAMP mice using a new scoring method that incorporates objective MRI radiomic analysis and pathology to accurately characterize disease in the small intestine. SAMP mice with established SI inflammation were found to exhibit MRI features of human CD including SI wall thickening, strictures, free fluid collections, and enlarged mesenteric lymph nodes (Fig. 4f). MRI images from SAMP were annotated by a blinded radiologist (Fig. 4g) and radiomic features were extracted (Fig. 4h). Radiomic feature selection was performed using imaging samples from the DEX (low disease severity) and PBS (high disease severity) groups (Fig. 4i). Features with significant separation between these groups were identified utilizing the Wilcoxon rank sum. The topmost discriminatory radiomic features were used to train a Random Forest predictive model which provided a probability of a high-severity sample (Fig. 4j). This model was then applied to the hMSCs-treated and additional PBS and DEX-treated groups. There was a positive correlation between MRI inflammatory scores and pathology scores (Supplementary Fig. 7). Combining radiomics score with pathology score (SIMPle score) resulted in further enhancement of disease severity assessment and discrimination between groups, with hMSC-treated mice demonstrating radiologic healing (PBS:34.21 ± 4.70 vs hMSC:26.5 ± 6.31; *P* = 0.0246, PBS:34.21 ± 4.70 vs DEX: 12.2 ± 7.13; *P* < 0.0001) (Fig. 4k). Overall, our results demonstrate that while DEX is potent, its effect is transient, whereas hMSCs have a sustained effect at day 28.

### Single-cell RNA sequencing of mesentery identified a novel macrophage pattern of hMSCs-mediated response

**Fig. 5:**
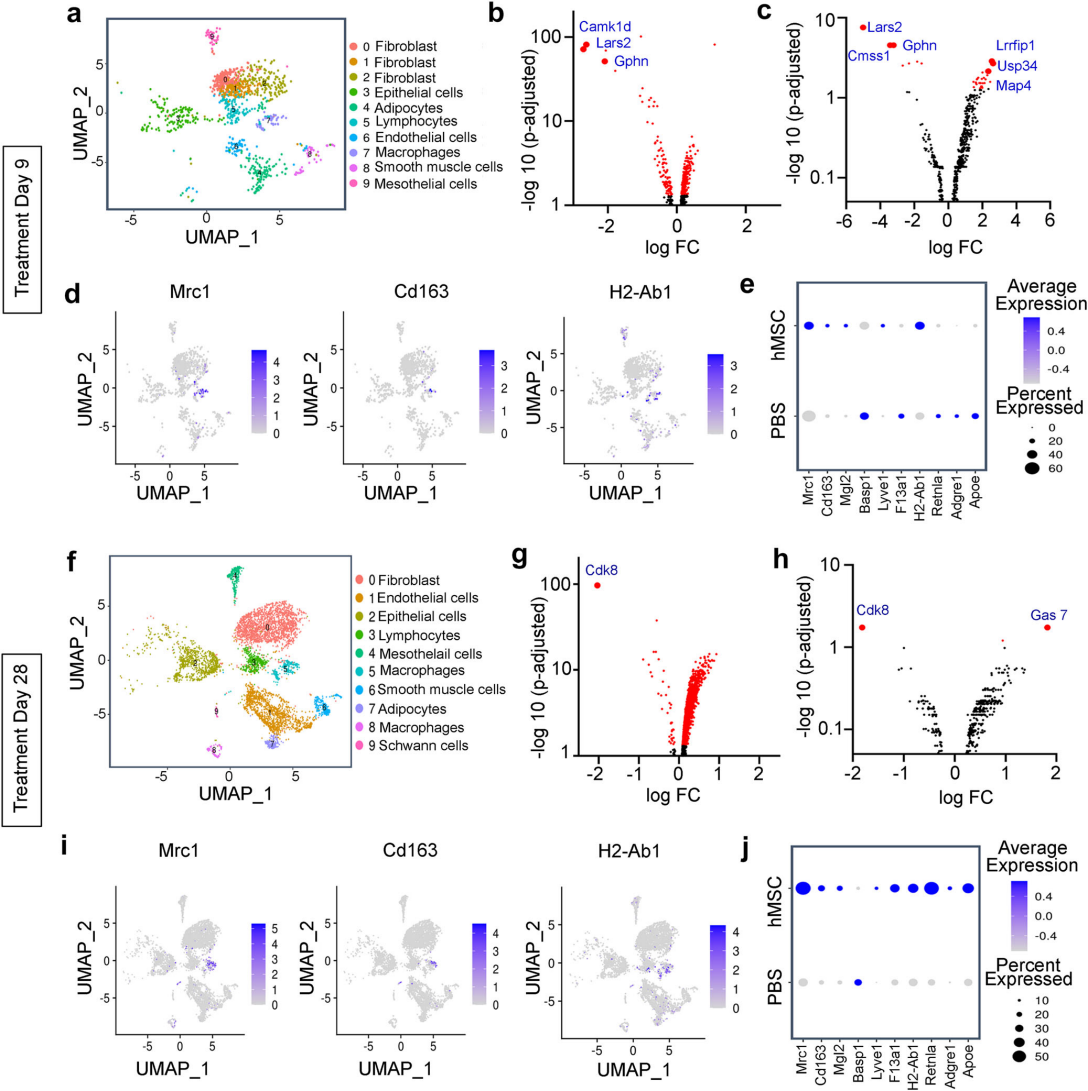
Sc-RNAseq was performed on single-cell suspension of mesenteric stromal vascular fraction (SVF) from SAMP mice treated with PBS and hMSC. **a–e** Sc-RNAseq showing analysis on day 9 samples. **a** UMAP plot showing identified clusters at a resolution of 1.0. **b** Volcano plots showing differential gene expression in all cell types with a threshold value (FDR < 0.05). The top three significant differentially expressed genes (logFC >1.5 with FDR < 0.05) are highlighted and labelled in the plots. **c** Volcano plots showing differential gene expression in macrophage cluster with a threshold value (FDR < 0.05). The top 3 significant differentially expressed genes (logFC>1.5 with FDR < 0.05) (upregulated and downregulated) are labelled and highlighted in the plot. **d** UMAP plots of the representative macrophage markers genes (*Mrc1, Cd163, H2-Ab1*) showing normalized gene expression in the macrophage cluster. **e** Dot plots showing the average expression of selective genes expressed by the proportion of the cells in the macrophage cluster across the treatment. **f**–**j** Single-cell RNA sequencing showing analysis on day 28 samples**. f** UMAP plot showing identified clusters at cluster resolution 0.4. **g** Volcano plots show differential gene expression in all cell types with a threshold value (FDR < 0.05). *Cdk8* was significant differentially expressed gene (logFC >1.5 with FDR < 0.05) in hMSC-treated mice on day 28. **h** Volcano plots showing differential gene expression in macrophage cluster with a threshold value (FDR < 0.05). Two genes were significantly differentially expressed (FC > 1.5 with FDR < 0.05) in hMSCs-treated mice. **i** UMAP plots of the representative macrophage markers genes (*Mrc1, Cd163, H2-Ab1*) showing normalized gene expression in the macrophage cluster. **j** Dot plots showing average expression of selective genes and proportion of the cells representing in the cluster across the treatment.

Our imaging data showed hMSCs on the mesentery of SAMP mice (Fig. 2d) and given increasing recognition of the importance of mesenteric inflammation in the pathophysiology of Crohn’s disease, we performed Sc-RNAseq on single cell suspension from the stromal vascular fraction (SVF) of mice mesentery on day 9 and day 28 of hMSCs treatment using SPLiT-seq method. Raw sequencing data were processed to qualify the quality criteria (Supplementary Fig. 8). Transcripts from mice treated with hMSCs at day nine clustered into ten groups at clustering resolution 1.0 with variable numbers of cells and UMI counts per cell (Fig. 5a). These clusters were annotated to fibroblasts, macrophages, T Lymphocytes, epithelial cells, mesothelial cells, smooth muscle cells, and Schwan cells using scMRMA and after manual curing based on available literature on cell types in Sc-RNAseq. DEG analysis performed on all cell types revealed a highly enriched population of differentially expressed genes in hMSC-treated mice (FDR < 0.05) with five genes (*Camk1d, Lars2, Xist, Gphn, Gm19951*) reaching statistical significance (Fig. 5b). As macrophage constitutes a major component of the mesentery and our in vitro data on macrophage polarization to anti-inflammatory phenotype, we studied in-depth the transcriptomic profile of the macrophage cluster. We observed larger numbers of differentially expressed genes in macrophage clusters in hMSCs-treated mice with 33 genes meeting statistical significance, among which *Lars2, Cmss1, and Gphn*, were the top three downregulated genes while *Map4, Usp34*, and *Lrrfip 1* genes were upregulated after hMSC treatment (Fig. 5c). *Mrc1*^27^*, Cd163*^28^*, H2-Ab1*^29^ are marker genes that are highly expressed in macrophages and were used to represent macrophage clusters in the UMAP plot (Fig. 5d). Based on published studies^30,31^ and heterogeneity in macrophage phenotype, we further analyzed a set of genes (*Mrc1, Cd163, Basp1, Mgl2, Lvye1, Retnla, Adgre1, Apoe, F13a1*)^32–36^ that represents M1 pro-inflammatory and M2 anti-inflammatory phenotype of macrophages in different tissues. Analogous to our in vitro data, we observed a marked increase in the average gene expression of genes representing M2 anti-inflammatory macrophages *(Mrc1, Cd163, Mgl2, H2-Ab1)* in the hMSC treatment group, however, the percentage of cells expressing these genes did not change across treatment (Fig. 5e).

Similarly, we have analyzed the therapeutic effect of hMSCs treatment at day 28 on the cellular composition and transcriptomic alterations in all detected cell types. The detected transcripts from PBS and hMSCs-treated SAMP were clustered into ten groups on UMAP plots (Fig. 5f) and annotated to fibroblast, macrophages, T Lymphocytes, epithelial cells, mesothelial cells, smooth muscle cells, and Schwann cells using scMRMA method and manually cured. DEG analysis on all cell types revealed a highly enriched population of differentially expressed genes in the hMSC group, with the *Cdk8* gene demonstrating statistical significance (Fig. 5g). DEG analysis in the macrophage cluster revealed several DEGs in the hMSC group, with downregulated *Cdk8* (kinase involved in inflammatory responses) and upregulated *Gas7* (gene involved in phagocytic cup formation in macrophage) genes meeting statistical significance criteria (Fig. 5h). Similar to the UMAP plot of day 9 samples, *Mrc1, Cd163, and H2-Ab1* marker genes represent the macrophage population on UMAP plots on day 28 samples (Fig. 5i). Lastly, we studied average gene expression and percent representation of cells expressing a set of genes in macrophage clusters using dot plot analysis. We found that hMSCs treatment resulted in a pronounced increase in the average gene expression of anti-inflammatory M2 phenotype *(Mrc1, Cd163, Mgl2, Lyve1, F13a1, Retnla, and Apoe)* along with an increase in anti-inflammatory macrophage numbers compared to the early response on day 9 (Fig. 5j).

### hMSCs educate macrophages to anti-inflammatory phenotype and suppress T cell proliferation to alleviate small intestine inflammation in SAMP

**Fig. 6:**
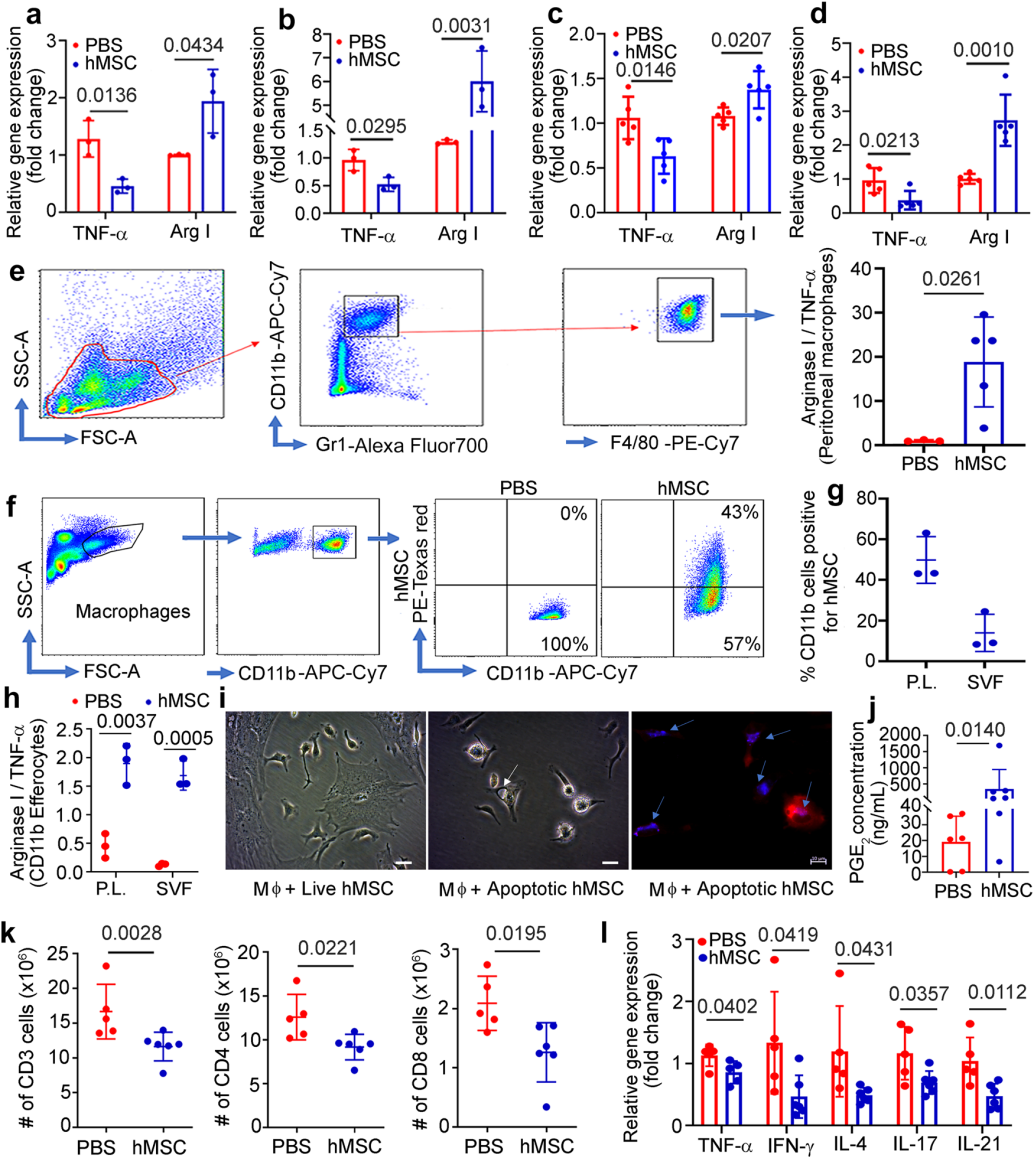
hMSC secrete PGE_2_, educates mLN, mesenteric, and peritoneum macrophages to anti-inflammatory phenotype, and suppresses T cells to alleviate small intestine inflammation in SAMP. Relative gene expression of TNF-α and Arginase I on day 9 of the treatment, measured in the total RNA extracted from CD11b^+^ cells from (**a**) mesenteric lymph node and **(b)** mesenteric stromal vascular fraction. **c**, **d** Relative gene expression of TNF-α and Arginase I on day 28 of the treatment measured in the total RNA extracted from CD11b^+^ cells from (**c**) mesenteric lymph node (**d**) mesenteric stromal vascular fraction. Gene expression was determined by qRT-PCR, normalized to GAPDH, and expressed as fold change (2-ΔΔCt). **e** Arginase 1/ TNF-α ratio measured by flow cytometry in SAMP peritoneal macrophages. **f, g** Cell Tracker Red^TM^ CMPTX dye labelled hMSCs showing in-vivo phagocytosis by CD11b^+^ macrophages in peritoneal lavage (P.L.) and SVF at day 9. **h** Arginase 1/ TNF-α ratio measured by flow cytometry in SAMP peritoneal and SVF Cd11b^+^ macrophages (Efferocytes) phagocytosed hMSC. **i** Brightfield microscopic images of SAMP peritoneal macrophages co-cultured with live hMSCs (no visible apoptosis observed) and apoptotic hMSCs (white arrow showing phagosome formation in macrophage), fluorescence image showing phagocytosis of apoptotic hMSCs by SAMP peritoneal macrophages, arrows showing engulfed apoptotic bodies of hMSC. Scale bar 10 µm. **j** PGE_2_ concentration measured in the peritoneal fluid of SAMP. **k** Absolute numbers of CD3, CD4, and CD8 lymphocytes measured in mLN at day 28 of the treatment. **l** Relative gene expression of cytokines in CD4 lymphocytes from hMSCs-treated SAMP at day 28. Data were expressed as mean ± SD from at least two independent experiments, each data point represent one mouse. *P* < 0.05, considered significant, by 2-tailed, unpaired Student’s *t* test.

We further confirmed the immunomodulatory effect of hMSCs on CD11b^+^ macrophages isolated from mLN, mesentery, and peritoneal macrophages at day 9 and day 28 after hMSCs treatment. At day 9, hMSCs treatment resulted in the anti-inflammatory phenotype of mLN and SVF macrophages with decreased gene expression of proinflammatory cytokines TNF-α and an increase in M2 anti-inflammatory phenotype marker Arginase-I (Fig. 6a, b). CD11b^+^ macrophages isolated from mLN and SVF of mesentery at day 28 also demonstrated similar anti-inflammatory phenotype with an increase in gene expression of surface marker Arginase-I and a decrease in gene expression of proinflammatory cytokines TNF-α (Fig. 6c, d). Additionally, we studied gene expression of several other proinflammatory cytokines including IL-1α, IL-1β, IL-12, and IL-6 at day 9 and day 28 of the treatment and most were downregulated after hMSCs treatment (Supplementary Fig. 9). Furthermore, we performed flow-based cell sorting of peritoneal macrophages Gr1, F4/80 and demonstrated that peritoneal macrophages from SAMP mice treated with hMSCs had an anti-inflammatory phenotype with higher Arginase-I/TNF-α ratio (*P* = 0.0261) (Fig. 6e). As no live hMSCs were detected at day 28 and given upregulation of *Gas7*, a marker of phagocytosis at day 28, we next assessed the ability of SAMP macrophages to phagocytose hMSCs via efferocytosis and therefore acquire anti-inflammatory phenotype^37^. Hence, we injected cell tracker Red^TM^ CMTPX dye-labeled hMSCs in SAMP and noted that CD11b+ macrophages efferocytose apoptotic hMSCs in the peritoneal cavity and mesenteric SVF (Fig. 6f, g) and these macrophages have anti-inflammatory phenotype with significantly higher Arginase-I/TNF-α ratio (Peritoneal lavage (P.L.); *P* = 0.0037, SVF; *P* = 0.0005) (Fig. 6h). Furthermore, our in vitro co-culture assay with live and apoptotic hMSCs demonstrated that SAMP macrophages did not show efferocytosis with live hMSCs while they show marked uptake of apoptotic hMSCs with the visible formation of the phagosome. We confirmed our results by labeling apoptotic hMSCs with annexin to mark compromised cell membranes, and these marked apoptotic hMSCs were detected in the F4/80 tagged peritoneal macrophages on co-culture (Fig. 6i).

**Fig. 7:**
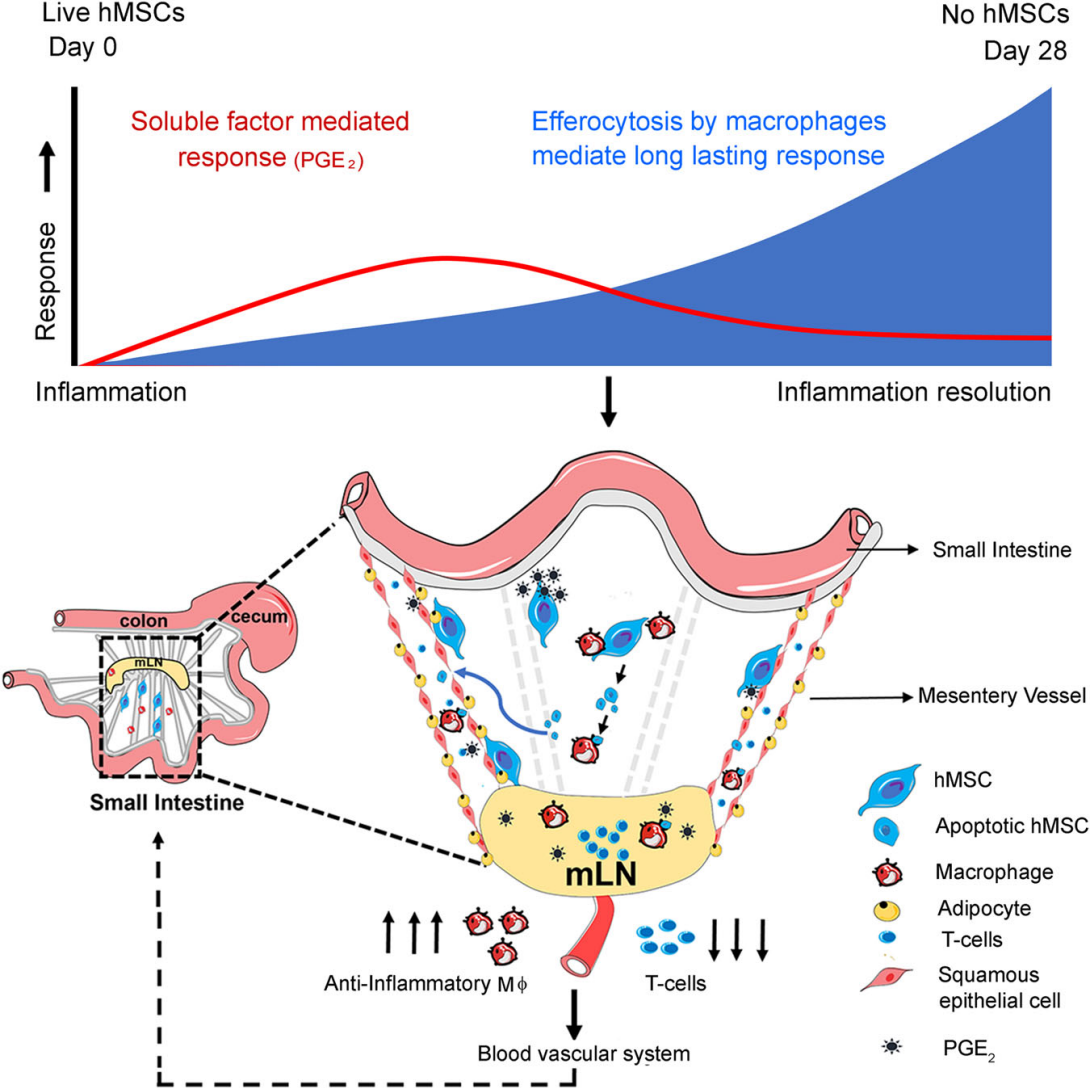
Schematic showing putative mechanism of hMSCs mediated immunosuppression in experimental model of Crohn’s disease. In the early stage, live hMSC secretion of molecules like PGE_2_ is the dominant mechanism. PGE_2_ is released by hMSCs in the peritoneal cavity which diffuses through the fenestrated mesenteric lymphatic vessels to reach mLN, inhibit naïve T cell proliferation and educate SVF and mLN resident macrophages to anti-inflammatory phenotype with subsequent downregulation of proinflammatory cytokines, and enhance macrophages efferocytosis capacity. In the later phase, macrophages efferocytose apoptotic hMSCs, which leads to their proliferation and reprogramming to an anti-inflammatory phenotype that maintains and mediates the long-term effect.

In addition to efferocytosis, we detected a significantly higher concentration of PGE_2_ (341.1 ± 605.6 ng/mL; *P* = 0.0140) in SAMP peritoneal fluid after intraperitoneal injection of hMSCs (Fig. 6j). Similar to our in-vitro data which demonstrated suppression of T cell proliferation by PGE_2_ secretion by hMSCs, we found that hMSCs resulted in T cell suppression in mLN with significant decrease in absolute numbers of CD3(PBS:16.65 ± 3.91; hMSC:11.63 ± 2.06, *P* = 0.0228), CD4(PBS:12.59 ± 2.60, hMSC:9.16 ± 1.45, *P* = 0.0221) and CD8 cells (PBS: 2.09 ± 0.45, hMSC:1.26 ± 0.50, *P* = 0.0195) (Fig. 6k) and CD4 cells show a decrease in gene expression of pro-inflammatory Th-1 (TNF-α; 0.85-fold change, *P* = 0.0402, IFN-γ; 0.46-fold change, *P* = 0.0419), Th-2(IL-4; 0.49-fold change, *P* = 0.0431), and Th-17 (IL-17;0.69-fold change, *P* = 0.0357, IL-21;0.47-fold change, *P* = 0.0112) cytokines (Fig. 6l). Briefly, hMSCs secrete PGE_2_ in the peritoneal cavity and lymphatic vessels to suppress T cell proliferation and reprogram macrophages to anti-inflammatory phenotype, and enhance macrophages’ efferocytosis capacity^38^. Peritoneal and SVF macrophages perform efferocytosis of apoptotic hMSCs and become polarized to anti-inflammatory phenotype followed by clonal expansion, which results in long-term efficacy (Fig. 7).

## Discussion

MSCs are a novel therapeutic approved in the EU, Japan, and Israel for the treatment of CD perianal fistulas and in advanced stage clinical trials for immune-mediated diseases like CD^7^, however, their mechanism of action in CD is unclear. Here, we show that hMSCs have potent immunosuppressive activity in vitro and result in histologic, mucosal, and radiologic healing of chronic intestinal inflammation in a spontaneous model of Crohn’s disease that closely resembles human disease. Following intraperitoneal administration, hMSCs did not home to the small intestine and mediated their effect via modulation of T cells and macrophages in the mesentery and mesenteric lymph node. Furthermore, our results demonstrate that while a steroid like dexamethasone is potent, its effect is transient, whereas hMSCs have prolonged effects that outlast their presence.

Using multiple imaging modalities and flow cytometry, we demonstrated that after intraperitoneal injection majority of hMSCs did not migrate to the small intestine and remained in the peritoneal cavity adjacent to the mesentery and lymph node. Our data is analogous to previous studies that show limited homing of MSCs to the colon after intraperitoneal administration in colitis models^22^. However, all detection techniques have sensitivity limitations and a smaller percentage of MSCs may not have been detected, therefore, we cannot rule out the possibility that a small percentage of hMSCs may have migrated to the small intestine or differentiated into the functional epithelial or stromal cells and contributed to the efficacy. As a very small fraction of hMSC survived beyond day 9 of administration, we studied hMSCs therapeutic effect and mechanism at day 9 and noted mucosal healing and immunological response. Since most hMSCs did not migrate to the small intestine, their secretory product including PGE_2_^39^ likely diffused through the fenestrated lymphatic vessels^40,41^ to access mesentery and reach mLN through the lymphatic drainage. These secretory products suppressed T cell proliferation and educated macrophages to anti-inflammatory phenotype, and this was supported by several lines of in-vitro and in-vivo evidence including; hMSCs secretion of PGE_2_, reduction of hMSCs effect on T-cell response by pharmacological blockade of PGE_2_, a higher concentration of PGE_2_ in SAMP peritoneal fluid after intraperitoneal injection of hMSCs, reduction in absolute numbers of CD3^+^ and CD4^+^ T cells in SAMP mLN with downregulation of gene expression of Th-1 and Th-2 cytokines, and conversion of macrophages to an anti-inflammatory phenotype in-vitro and in mLN, mesenteric and peritoneum macrophages of SAMP, and macrophage efferocytosis. We further characterized hMSCs therapeutic mechanism by unbiased Sc-RNAseq on SVF mesenteric cells and noted hMSCs treatment resulted in differential expression of genes across all detected cell types in SVF. Mesenteric inflammation is implicated in Crohn’s disease, however, no medical therapy specifically targets mesenteric inflammation^42–44^. Our study shows that mesentery could be targeted for the treatment of small bowel inflammation.

Sc-RNAseq provided further evidence of macrophage polarization to anti-inflammatory phenotype as hMSCs downregulated the expression of several genes involved in inflammatory pathways in macrophages. hMSCs treatment upregulated gene expression of transcriptional repressor *Lrrfip1* in macrophages, which codes for the Leucine-rich repeat flightless-interacting protein 1 and directly binds to the GC-rich sequences of the tumor necrosis factor-alpha (TNF-α) promoter and inhibit its production^45^. We also observed an increase in the average expression of a set of genes (*Mrc1, Cd163, Mgl2, Lyve1, F13a1, Retnla, and Apoe*), representing heterogeneous tissue resident anti-inflammatory M2 macrophages in the mesentery of hMSCs treated SAMP, however, number of these macrophages did not change across treatment groups by day 9.

At day 28, hMSCs treatment results in a complete response in SAMP mice marked by mucosal, histological, immunologic, and radiological healing. Immunologically, there was a reduction in absolute numbers of CD3^+^, CD4^+^ T, and CD8^+^ T cells in SAMP mLN with downregulation of gene expression of multiple Th-1 and Th-2 cytokines, and an increase in anti-inflammatory macrophages in mLN, mesenteric and peritoneum macrophages of SAMP treated with hMSC. The increased time required for a complete response is likely due to the significant histologic inflammation in SAMP mice with established disease that require a longer time to heal as well as the unique mechanism of action of hMSCs. Our day 28 Sc-RNAseq data showed downregulation of *Cdk8* and upregulation of *Gas7* genes in macrophages and demonstrated an increase in the number of anti-inflammatory macrophages compared to day 9. Interestingly, these genes were not DEGs in macrophages during the early phase, indicating the dominance of this mechanism in the mesenteric macrophages in the absence of live hMSCs. *Gas7* is involved in the development of the phagocytic cap and is highly expressed in macrophages capable of phagocytosis^46^. Efferocytosis, a process of clearing dead and apoptotic bodies by phagocytic cells, can induce proliferation in macrophages and result in an anti-inflammatory phenotype^37^. Our data show that SAMP macrophages phagocytose hMSCs in the peritoneal cavity and result in an anti-inflammatory phenotype. A significant percentage of hMSC apoptotic bodies are uptaken by SVF resident macrophages, which resulted in a marked increase in numbers of heterogeneous SVF resident anti-inflammatory macrophages with higher expression for *Mrc1, Cd163, Mgl2, Lyve1, F13a1, Retnla*, and *Apoe* genes at day 28. Our data is congruent with a study that used clinical samples from patients with graft-versus-host-disease treated with MSCs and murine models to show that MSCs undergo apoptosis after infusion, and this is the primary driver of their immunosuppressive function and clinical response in patients^47^.

In summary, MSCs are short-lived after administration and yet can have prolonged therapeutic effects, which have been unexplained. Our results demonstrate that hMSCs modulate the immune response in SAMP in stages. In the early stage, live hMSCs secretion of molecules like PGE_2_ is the dominant mechanism. These molecules released by hMSCs in the peritoneal cavity diffuse through the fenestrated mesenteric lymphatic vessels to reach mLN, inhibit naïve T cell proliferation and educate SVF and mLN resident macrophages to anti-inflammatory phenotype with subsequent downregulation of proinflammatory cytokines, and enhance macrophages efferocytosis capacity. Secretory molecules like PGE_2_ work universally and induce immunosuppression across the species as demonstrated by our in vitro MLR and macrophage co-culture data. In the later phases following hMSC treatment, macrophages efferocytose apoptotic hMSCs which leads to their proliferation and reprogramming to anti-inflammatory phenotype which is maintained and mediates the long-term immune effects. Meriwether et al. showed that after efferocytosis, macrophages upregulate Cox2 and PGE_2_ production^38,48^. which then led to increased activity of Rac1, and subsequent increase in the numbers of macrophages efferocytosing apoptotic cells^48^. Metabolites produced from apoptotic cells after efferocytosis have been demonstrated to reprogram macrophages in multiple ways^49,50^. Yurdagul et al. demonstrated that arginine produced in macrophages after efferocytosis promotes continual efferocytosis via HuR protein-mediated stabilization of *Mcf2* mRNA that facilitates Rac1 activation and leads to resolution of injury in an atherosclerosis model^50^. Furthermore, Ampomah et al. demonstrated that apoptotic cell-derived methionine induces the activity of DNA methyltransferase 3A (DNMT3A) that induces epigenetic modification that represses *DUSP4* function, allowing for uninterrupted ERK signaling and production of PGE_2_ and TGFβ^49^. In our study, these anti-inflammatory macrophages suppress the proliferation and numbers of potentially pathogenic T cells in mLN resulting in reduced numbers in the small intestine and contributing to the marked reduction in small intestine inflammation.

Our study has a few limitations, including the employment of human MSCs in an immune-dysregulated mouse. Human and mouse MSCs are considered immune-evasive and characteristically have low expression of MHC II, CD40, CD80, and CD86^51,52^, and multiple prior studies have utilized human MSCs to ameliorate inflammation in murine models of acute colitis and did not find any induction of immunological response^15,53^. In addition, the mechanism of action that demonstrates PGE_2_ is an important molecule that induces immunosuppression across the species; and macrophage phagocytosis of apoptotic hMSC partly obviates these concerns. Our in-vitro data suggests that PGE_2_ is a major contributor but did not fully account for MSC-mediated immunosuppression. In addition to PGE_2_, MSCs exert immunosuppressive effects through multiple mechanisms, including secretory molecules like Tumor necrosis factor stimulated gene-6 (TSG-6)^22^, exosomes^54^, and contact-dependent factors like PD-1 pathway^55^. A proteomic analysis of MSCs and their secretome may provide additional insight into other factors contributing to MSC-mediated immunosuppression and will be investigated in future studies. Another limitation is that a small percentage of MSCs may have migrated to the small intestine which is undetectable by our current methods.

hMSCs are short-lived which contributes to their safety profile noted in human clinical studies but exert immunosuppressive and tissue regenerative properties that outlast their presence in the body, our study provides a mechanism for this observation. Mesenteric inflammation is implicated in Crohn’s disease, however, no medical therapy specifically targets mesenteric inflammation. Our study shows that mesentery could be targeted for the treatment of small intestinal inflammation via MSCs and shows multiple potential avenues for enhancing the potency of MSCs via modulation of their secretome/PGE_2_ and/or their efferocytosis ability, which can then be tested for efficacy in SAMP murine models.

## Methods

### Mice

SAMP1/YitFc (SAMP) mice were obtained from Case Western Reserve University (CWRU) under the material transfer agreement and rederived at UC Davis. Initial experiments on SAMP mice were performed at Case Western Reserve University [CWRU; IACUC (Institutional Animal Care and Use Committee) protocol # 2015-0142] and then with SAMP mice housed at UC Davis vivarium. The animals were housed in AAALAC (Association for Assessment and Accreditation of Laboratory Animal Care) approved specific pathogen-free facility of the UC Davis Institute of Regenerative Cures, UC Davis Health, Sacramento, and maintained in Tecniplast IVC (Individually Ventilated Cages) blue line, following the standard 12-h light/dark cycles. All mice had ad libitum access to water and were fed the standard global 18% protein rodent diet throughout the experiments. All experimental procedures were approved by the Institutional Animal Care and Use Committee of UC Davis (IACUC protocol # 21298). Both male and female mice with established disease (>24 weeks old) were randomly assigned to the control and treatment groups to minimize the confounders. All outcome assessments of the experiments including histopathology scoring, stereomicroscopy, RT qPCR, radiomics, and MRI scoring were performed in a blinded manner.

### Bone marrow-derived human mesenchymal stem cells (BM-hMSCs) isolation and expansion

Bone marrow was collected from healthy de-identified donors after informed consent; the procedure was reviewed and approved by the University Hospitals of Cleveland and the University of California Davis Institutional Review Boards. The hMSCs were isolated as per previously described protocols^56,57^. Briefly, isolated bone marrow was mixed with DMEM-LG + 10%FBS medium and sample were centrifuged at 450 *g* for 5 min. Cell pellets were processed using Percoll gradient method to remove RBCs and collect nucleated cells were resuspended in DMEM-LG + 10%FBS medium. Cell suspension volume was adjusted to seed the cells at 1.8 × 10^5^ nucleated cells per cm^2^ of culture vessels and cultured for 14 days; culture medium changed every 3 to 4 days. Finally, cells were harvest using trypsin and further sub-cultured DMEM-LG + 10%FBS medium for multiplication. The hMSCs were successfully stored at P3 in liquid nitrogen and subsequently expanded in a T175mm^2^ tissue culture flask using DMEM, selected lots of 10% FBS supplemented with 2mM L-glutamine and 1% penicillin-streptomycin before injection. The cells were maintained in the CO_2_ incubator at 37 °C and 5% CO_2_ for 9–11 days, culture media was changed every 3–4 days.

### Fibroblasts isolation and expansion

Human skin fibroblasts were isolated as a previously described method^58^. Briefly, human skin, keratomed at a depth of 0.4 mm from the buttocks region of volunteers, was provided by the Skin Diseases Research Center, Case Western Reserve University School of Medicine, under an approved IRB protocol. The epidermal and dermal components were separated by treatment with 50 U/ml dispase (Collaborative Biomedical Products, Bedford, Mass.) overnight at 4 °C. The dermal component was minced and incubated at 37 °C for 1 h with an enzyme cocktail consisting of 100 mM sodium pyruvate, 0.1 mg/ml DNase, 1.25 mg/ml hyaluronidase, and 2.7 mg/ml collagenase C in DMEM-LG (Dulbecco’s Modified Eagle Medium-low glucose) buffered with HEPES, pH 7.4. The resulting tissue suspension was filtered through 100-μm nylon meshes to separate single cells and small aggregates from larger pieces of tissue and cultured in DMEM-LG supplemented with 10% FBS.

### Chondrogenic differentiation

Cells were trypsinized and then resuspended in a chondrogenic differentiation medium (DMEM-high glucose supplemented with 1% Insulin-Transferrin-Selenium (ITS) + 10^-7 ^M dexamethasone, 1 mM sodium pyruvate, 120 mM ascorbic acid-2 phosphate, 100 mM nonessential amino acids, and 10 ng/mL TGF-β1). Two hundred microliters of this cell suspension containing 250,000 cells were added per well of a 96-well polypropylene V-bottom, multiwell dish (Phenix Research). The multiwell plates were centrifuged at 500 *g* for 5 min and then incubated at 37 ^o^C. The differentiation medium was changed every other day. Chondrogenic pellets were harvested after 21 days for histological analysis.

### In vivo osteogenesis

In vivo osteogenesis of MSCs is tested by their capability of forming ectopic bone tissue. Briefly, cells are vacuum-loaded into fibronectin-coated 3 × 3 × 3 mm hydroxyapatite/tricalcium phosphate cubes generously provided by the Zimmer Corporation (Warsaw, IN). Cell-loaded cubes were then incubated at 37 °C for 2 h in a humidified atmosphere of 5% CO_2_ and 95% air. After the incubation period, individual cubes were implanted subcutaneously on the dorsal surface of severe combined immunodeficient mice following an approved IACUC protocol. Animals were euthanized after 6 weeks, and the cubes were fixed with 10% phosphate-buffered formalin.

### Cell transduction

hMSC were seeded at 1 × 10^4^ cells per cm^2^ and transduced with a multiplicity of infection (MOI) of 5 in the presence of protamine sulfate and transduced for 24 h. Three days after the last round of transduction, the efficiency is measured by detecting the red fluorescent protein (RFP) by flow cytometry. To evaluate that the transduction process does not affect the stem cell properties of MSCs, the chondrogenic (in vitro) and osteogenic (in vivo) capabilities were assessed.

### Mixed lymphocyte reaction (MLR)

Mixed lymphocyte reaction was performed as per our previously published methodology^59^. Briefly, splenocytes were isolated from two different strains of mice (C57BL/6J and SAMP/YitFc). One set of splenocytes served as responder cells, while the other set was irradiated at 3300 Gy and served as stimulator cells. Briefly, 1 × 10^6^ activated mixed splenocytes [SAMP/YitFc(responders)+C57BL/6(stimulator)] were co-cultured with the irradiated human mesenchymal stem cells (3300 Rad for 10 min) in different cell ratios (1:20, 1:10,1:5) in microwell plates for 4 days. Human dermal fibroblast was taken as a cell therapy control. The suppression of the T-cells was studied after adding the ^3^H-Thymidine (PerkinElmer cat#NET027W001MC) on day 4. The plates were incubated for another 18 h in a radioactive incubator during which ^3^H-thymidine will be incorporated into the newly synthesized DNA of the dividing cells. The proliferation was calculated by measuring the uptake of [3H] thymidine in a scintillation counter and is expressed as counts per million.

### Macrophage and hMSC co-culture

SAMP bone marrow was harvested and enriched for CD14 cells using CD14 MACS purification kit (Miltenyi Biotec cat# 130-100-629). CD14 monocytes were further cultured in complete RPMI1640 with 50 ng/ml MCSF (Macrophage colony-stimulating factor) for 7 days, culture media was changed every 3rd day with fresh MCSF (Peprotech, cat#315-02). After 7-day culture, CD14 cells were harvested and cultured with hMSC in a 1:1 ratio for 48 h. Reaction supernatant was collected to estimate PGE_2_ concentrations using ELISA and cells were processed to isolate total RNA for gene expression analysis.

### Prostaglandin E2 (PGE_2_) enzyme immunoassay

MLR and macrophage co-culture reaction supernatant were collected and stored at −80 °C for PGE_2_ analysis. For the in vivo estimation of PGE_2_ in mice, 10 ml of PBS was injected into the peritoneum cavity of mice and peritoneal lavage was performed to collect the peritoneal fluid. Peritoneal fluid was centrifuged at 300 × *g*, and supernatant was collected and stored at −80 °C for PGE_2_ analysis. PGE_2_ was analyzed using the previously published methodology^59^ following the manufacturer protocol (Cayman Chemicals, cat# 514010). The assay is a competitive immunoassay in which prostaglandin E_2_ compete with PGE_2_-acetylcholinesterase conjugate (PGE_2_-tracer) for the limited amount of PGE_2_-monoclonal antibody. The samples were incubated for 18 h at 4 °C followed by addition of Ellman’s reagent, which served as the substrate for acetylcholinesterase. The proportion of the bound tracer was determined spectroscopically, and the concentration of PGE_2_ was calculated using standard curves from known concentrations.

### hMSC treatment in SAMP

SAMP-1/YitFc mice with established inflammation (>25 weeks) were used for the in vitro and in vivo studies. In vitro cultured hMSCs (5.0 × 10^6^) were resuspended in 200 µl PBS and intraperitoneally injected in mice. An equivalent dose of 200 µl PBS served as vehicle control, while a daily dose of dexamethasone (2 mg/kg of mice weight) for 7 days was used as a positive control. Mice were euthanized using overdose of CO_2_ to effect followed by the cervical dislocation at the endpoints of the treatments.

### Stereomicroscopic examination

The distal ileum (10 cm) was fixed in Bouin’s solution (Sigma cat# HT1013) and imaged using a stereomicroscope (Amscope) with 10× optical zoom to score the inflamed areas. The tissue was imaged in 1 cm sections, followed by a complete image compilation. The area with cobble stoning and villus flattening was considered inflamed, and the percent disease area was calculated using the ImageJ software package.

### Tissue collection and histological evaluation

The distal ileum (10 cm) was fixed in Bouin’s solution, paraffin-embedded, cut into 5μm sections, and stained with hematoxylin/eosin. Histological assessment of tissues was performed by a trained pathologist, blinded to the treatment. A standardized semi-quantitative scoring system was followed as described in the study^24,60^. The scoring system involves the evaluation of 5 histologic parameters: (1) villus architecture (2) active inflammation (3) chronic inflammation (4) mononuclear infiltration (5) transmural inflammation.

### Gene expression analysis

Total RNA was extracted using RNeasy Mini Kit (Qiagen cat# 74106), following the manufacturer protocol, and quantified using a broad range Qubit 2.0 RNA Quantitation fluorometer kit (Thermo Fischer cat#Q10210). cDNA was synthesized using High-Capacity cDNA Reverse Transcription Kit with RNase Inhibitor (Applied Biosystem, cat#4374966). The primers were selected from the reported scientific literature; oligonucleotide sequences are shown in Supplementary Table 1. Real-time qPCR was performed in Bio-Rad thermocycler (Bio-Rad C1000 CFX384) using SsoAdvanced Universal SYBR Green Supermix (BioRad, cat# 1725272). GAPDH is used as the reference gene in the mRNA expression. The relative expression was obtained with BioradCFX Maestro 1.0 software and gene expression was determined using the standard curve method for each gene. The data are plotted as fold change using GraphPad Prism Version 9.0 (GraphPad Software, San Diego, CA).

### Flow cytometry

The mice were euthanized after the specified treatment period and mesenteric lymph nodes (mLN) were harvested in the complete T cell media (RPMI1640 + 10% FBS + 2 mM Glutamine + HEPES + NAA + Sodium pyruvate + β-mercaptoethanol). The mesenteric lymph nodes (mLNs) were processed to form a single-cell suspension using the syringe top and 70-micron strainer. Cells were harvested and counted using a hemocytometer. 1×10^6^ cells were first incubated with the Fc block (anti-CD16/32), and later surface staining was performed using fluorochrome-conjugated monoclonal antibodies purchased from BioLegend: APC-anti-CD45 (30-F11), BV785-anti-CD3(17A2), BV711-anti-CD4(RM4-5), BV605-anti-CD8α (53-6.7). Live/Dead^TM^ fixable aqua dead cell stain kit (Invitrogen) was used to stain the dead cells. For cell sorting, mouse peritoneum cavity was washed three times using ice-cold PBS (8 mL). Harvested cells were centrifuged at 300xg and stained with Alexa fluor700-anti-Gr1(BioLegend, RB6-8C5), PE/Cy7-anti-F4/80 (BioLegend, BM8) and APC-Cy7-anti-CD11b (BioLegend, M1/70). A population of cells positive for CD11b, Gr-1 and F4/80 were selected and sorted. Flow cytometry data were acquired on the LSR Fortessa flow cytometer (BD Biosciences, UC Davis Comprehensive Cancer Center, flow cytometry core, support by NCI P30CA093373) and analyzed using FlowJo 10.6.1 software (FlowJo, LLC).

### In vivo phagocytosis by CD11b macrophages

In-vitro cultured hMSCs were trypsinized and stained with CellTracker^TM^ Red CMTPX dye (Invitrogen, cat# C34552) as per the manufacturer’s protocol and injected peritoneal (5 million per mouse). Peritoneal lavage (P.L.) was performed on day 9 after injecting 10 ml of PBS in the peritoneal cavity, peritoneal fluid was centrifuged at 300 *g* for 5 min to collect the cells. Mouse mesentery tissue (stromal vascular fraction; SVF) was processed as per the previously reported method^61^. Single cell suspension from peritoneal lavage (P.L.) and SVF was later stained for APC/Cy7 anti-CD11 b monoclonal antibody (BioLegend, M1/70), PE-Cy7-anti Arginase-1 (Invitrogen, A1exF5) and APC-anti-TNF-α (BD Pharmingen^TM^, MP6-XT22).

### In vitro phagocytosis by peritoneal macrophages

Peritoneal lavage was performed in SAMP mice and peritoneal cells were collected after centrifugation at 300 *g* for 5 min. Collected peritoneal cells were further enriched for peritoneal macrophages using a MACS isolation kit (Miltenyi Biotec GmbHcat#130-110-434). An enriched population of macrophages was cultured on a glass coverslip in 6 well plates overnight in RPMI1640 medium. Live and apoptotic hMSCs were co-cultured with surface-adhered macrophages in a 1:1 ratio; hMSC were heat treated at 60 °C for 45 min to induce apoptosis and imaged at different time intervals to observe phagocytosis using a brightfield microscope. For the fluorescence imaging, apoptotic hMSCs were stained with annexin-Pacific Blue (BioLegend cat#640918) dye as per the manufacturer protocol and co-cultured with peritoneal macrophages for 2 h. After 2 h, cultured wells were washed three times with PBS, fixed using 4% formaldehyde for 20 min at 4 °C, and permeabilized using 0.1% tritonX100 for 15 min at room temperature (RT). Cells were further blocked for 30 min at RT using 1% BSA in PBS containing 0.1% tritonX100 and stained with PE/Cy-7 anti-F4/80 (BioLegend, BM8) as per manufacturer protocol. Cells were washed with PBS; glass coverslips were mounted on glass slides using 80% glycerol in PBS as mounting media and imaged using a fluorescence microscope (ZEISS).

### In vivo animal imaging

Bioluminescent imaging (BLI) was performed after the subcutaneous (SQ) injection of 150 mg/kg of luciferin substrate, using a Xenogen IVIS 200 series system. Fifteen minutes after intraperitoneal administration of bioluminescent hMSCs, an early BLI is performed to evaluate cell distribution throughout the mouse body. Later, images are taken every alternate day to evaluate cell localization. Additionally, hMSCs were tagged using a NIR dye IVISense680 (Perkin Elmer cat#NEV12001) following manufacturer protocol, and time course epifluorescence imaging was performed using IVIS Spectrum (Perkin Elmer). Following the removal of abdominal hair and verification of lack of fluorescence from intestinal contents, 5 × 10^6^ labeled hMSCs were peritoneally injected and live imaging was performed immediately on day 0 and followed on days 9 and 28. After completion of the experiment, colon, and mLN tissue were harvested and single-cell suspension was prepared to detect hMSCs using flow cytometry. Cells were stained using PE anti-human CD73(AD2), and APC anti-human CD105 (SN6) antibodies from eBioscience to detect hMSC.

### MR imaging

Twenty-six SAMP mice (34–40 wk) with established small instestine (SI) inflammation together with parental AKR control mice were imaged at the time of euthanasia in a 9.4 Tesla preclinical MRI scanner using the T2 fat suppression protocol in two independent experiments. To ameliorate SI inflammation, mice were injected with dexamethasone 2 mg/g/daily intra-peritoneally (i.p) for 7 days before euthanasia, and eleven mice with 5 million human mesenchymal stem cells (hMSC) i.p. 4 weeks prior to euthanasia. As disease control, mice were treated with PBS i.p. 4 weeks prior to euthanasia. A radiologist blinded to treatment annotated the small bowel in mice. Histopathology was performed on small intestine samples of all the mice to correlate disease severity with MRI scores. Radiomic analysis was conducted within annotated bowel wall regions on the four axial sections. From each section, 100 radiomic features from four different classes were extracted to quantify bowel wall appearance via different texture and image analytic operators to yield quantitative measurements of image heterogeneity and gradient responses. Statistical feature selection and pruning were conducted to identify the top radiomic features that could best differentiate between groups selected via statistical testing. Top-ranked radiomic features primarily comprised local neighborhood heterogeneity (*P* < 0.001) and filtered intensity responses (*P* < 0.001). These top-ranked features were used to train a machine learning classifier to yield a radiomics-based likelihood of disease severity for all SAMP mice (*n* = 26), that was scaled to range from 0 to 20. A Small Intestinal MRI Pathology Inflammation Score was computed for each mouse based on the summation of radiomic and histology scores.

### Single-cell RNA sequencing

Stromal vascular fraction (SVF) of mice mesentery was isolated using a previously reported method^61^. Briefly, mice were euthanized after completion of treatment, and mesenteries were precisely harvested in cold PBS. Mesenteric tissue was minced into small pieces and vortexed to collect SVF. SVF was further subjected to enzymatic digestion at 37 °C and 200 rpm for 30 min using digestion media (Collagenase-Type A, Deoxyribonuclease 1, 100 mM calcium chloride; sucrose, BSA, HEPES, PBS). The enzymatic digest was centrifuged at 300 *g* for 30 min at 4 °C and the pellet was resuspended in complete RPMI media and filtered through a 70 micron cell strainer. Single-cell RNA sequencing was performed using the SPLiTseq method from Parse Biosciences; cells were sequenced using the NovaSeq6000: S4(PE150) Illumine platform at the sequencing depth of 50,000 reads/cells. Log fold change >1.5 and FDR < 0.05 are considered statistically significant in differentially expressed genes (DEGs).

### cDNA library preparation, sequencing, and data analysis

Isolated single cells were counted using a hemocytometer and fixed using a cell fixation kit from Parse Biosciences. Fixed cells were counted using a hemocytometer before cDNA library preparation and library construction was completed using a Single Cell Whole Transcriptome-100k cells/nuclei kit from Parse Biosciences. 80,000 cells across 8 mice (*n* = 2 for day 9, *n* = 6 for day 28) samples were initially fixed using a cell fixation kit and later subjected to cDNA library preparation. In total ~50,000 cells were recovered after fixation and 5 sub-libraries were prepared. Two sub-libraries with ~20,000 cells were sequenced at 50k read, which includes ~3600 cells across two samples in the day 9 treatment and ~11,000 cells across six samples for the day 28 experiment. Cells with fewer than 200 genes, with mitochondrial gene expression greater than 20%, and with a number of UMIs below 200 or above 50000 were filtered. After completion of sequencing, raw sequencing data were deconvoluted to generate gene expression data for single cells, using Parse Biosciences’ data analysis pipeline (split-pipe v0.9.3) with default parameters and GRCm39 mouse reference genome. Gene expression data were preprocessed, clustered, and UMAP coordinates were calculated using Seurat, version 4.1.0^62^, running in R version 4.1.0^63^. Data were log normalized and corrected before clustering by regressing out a number of UMIs, percentage of mitochondrial gene expression, and cell cycle scores, using Seurat’s ScaleData function. Clustering resolutions of 0.4 and 1 were chosen to present clusters in day 9 and day 28 samples. Differential expression (DE) analyses were conducted on log normalized data using limma, version 3.50.3^64^. The model used in limma included effects for treatment, sample within treatment, and percent mitochondrial gene expression. DE analyses included genes expressed in at least 10% of cells out of all cells of a given type or included in each analysis.

### Statistics

All the animals were randomized. No statistical method was used to predetermine the sample size. The sample size of each experiment and statistical details are provided in the respective figure legend. The statistical analysis was performed using GraphPad Prism (version 9.0; GraphPad Software, San Diego, CA). The student’s two-tailed unpaired *t* test or Mann-Whitney test was used to compare the mean of two normally distributed groups. A one-way ANOVA test with Tukey as a posthoc test was applied for comparison between more than two groups. Data were expressed as mean ± SD. *P* values were considered statistically significant if *P* < 0.05, and each data point corresponds to a single mouse.

### Study approval

All experimental procedures were approved by the Institutional Animal Care and Use Committee of UC Davis (IACUC protocol # 21298) and Case Western Reserve University (CWRU; IACUC#2015-0142).

### Reporting summary

Further information on research design is available in the Nature Research Reporting Summary linked to this article.

### Supplementary information

Supplementary information

Reporting summary

## Data Availability

All raw sequencing data generated in this study have been deposited to the NCBI Sequence Read Archive (SRA) (https://www.ncbi.nlm.nih.gov/sra) under accession number PRJNA1052108.

## Code availability

The Codes for radiomic analysis are deposited on GitHub at https://github.com/ccipd/Radiomic_Mouse_Analysis.
