# Supplementary material for: MSCs mediate long-term efficacy in a Crohn’s disease model by sustained anti-inflammatory macrophage programming via efferocytosis
Source: NPJ Regen Med. 2024 Jan 20;9:6. doi: 10.1038/s41536-024-00347-1 (PMC10799947; doi:10.1038/s41536-024-00347-1)
Supplement: Supplementary file 1 — Supplementary information [file 41536_2024_347_MOESM1_ESM.pdf]

Supplementary Information

MSCs mediate long-term efficacy in a Crohn's disease model by sustained anti-inflammatory macrophage programming via efferocytosis

Maneesh Dave*[1,2], Atul Dev[1], Rodrigo A Somoza[3], Nan Zhao[4], Satish Viswanath[5], Pooja Rani Mina[1], Prathyush Chirra[5], Verena Carola Obmann[6], Ganapati H Mahabeleshwar[7], Paola Menghini[4], Blythe Durbin-Johnson[8], Jan Nolta[2,10], Christopher Soto[1], Abdullah Osme[7], Lam T Khuat[9], William J. Murphy[9,10], Arnold I Caplan[3], Fabio Cominelli [4]

Author Affiliations:

1 Division of Gastroenterology and Hepatology, Department of Internal Medicine, UC Davis Medical Center, University of California Davis School of Medicine, Sacramento, CA, United States

2 Institute for Regenerative Cures, University of California Davis School of Medicine, Sacramento, CA, United States

3 Skeletal Research Center, Department of Biology, Case Western Reserve University, Cleveland, OH, United States.

4 Division of Gastroenterology and Liver Disease, University Hospitals, Case Western Reserve University, Cleveland, OH, United States.

5 Department of Biomedical Engineering, Case Western Reserve University, Cleveland, OH, United States.

6 Department of Diagnostic, Interventional and Pediatric Radiology, Inselspital, Bern University Hospital, University of Bern, Switzerland.

7 Department of Pathology, School of Medicine, Case Western Reserve University, Cleveland, OH, United States.

8 Division of Biostatistics, Department of Public Health Sciences, University of California Davis School of Medicine, Sacramento, CA, United States.

9 Department of Dermatology, University of California Davis School of Medicine, Sacramento, CA, United States

10 Division of Malignant Hematology/Cell and Marrow Transplantation, Department of Internal Medicine University of California Davis School of Medicine, Sacramento, United States

***Correspondence:**

Maneesh Dave, MD, MPH
Associate Professor of Medicine
Division of Gastroenterology and Hepatology
Department of Internal Medicine
University of California Davis School of Medicine
Sacramento, CA, United States
4150 V Street, PSSB 3500, Sacramento, CA 95817
Tel: +1 916 734 7230
Email: mdave@ucdavis.edu

Conflict-of-interest: The authors have declared that no conflict of interest exists.

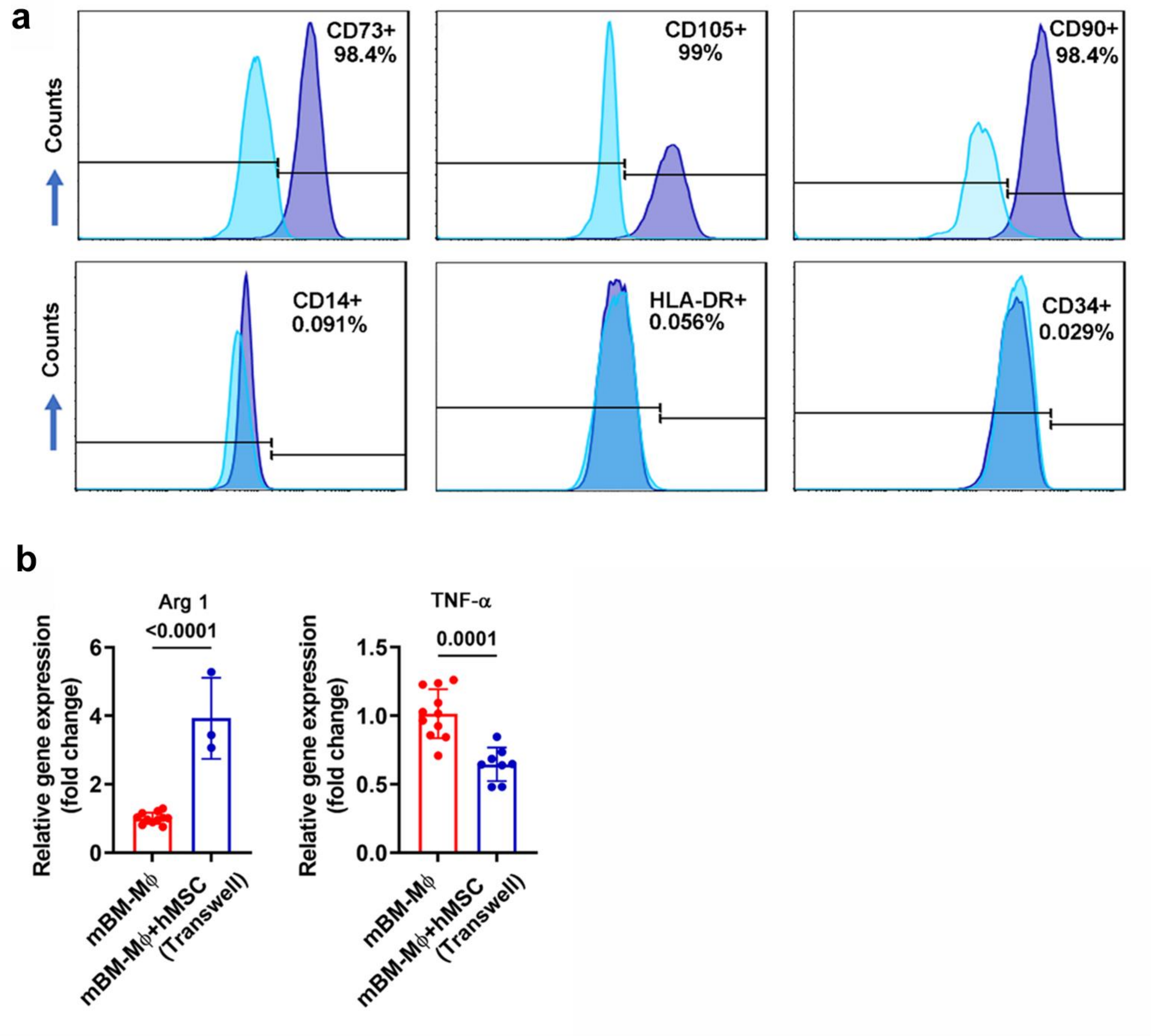

Supplementary Figure 1. Human bone marrow mesenchymal stem cells characterized by **(a)** positive CD73, CD105, CD90 and negative CD14, HLA-DR, and CD34 surface marker **(b)** Relative gene expression of Arginase-I (3.98-fold change, P<0.0001) and TNF-α (0.64-fold change, P<0.001), in total RNA extracted from murine bone marrow-derived macrophages co-cultured with hMSC (separated through trans well). The gene expression was determined by qRT-PCR, normalized to GAPDH, and expressed as fold change (2-ΔΔCt); Data represented as mean ±SD and correspond to at least two independent experiments, and each data point represents one mouse.

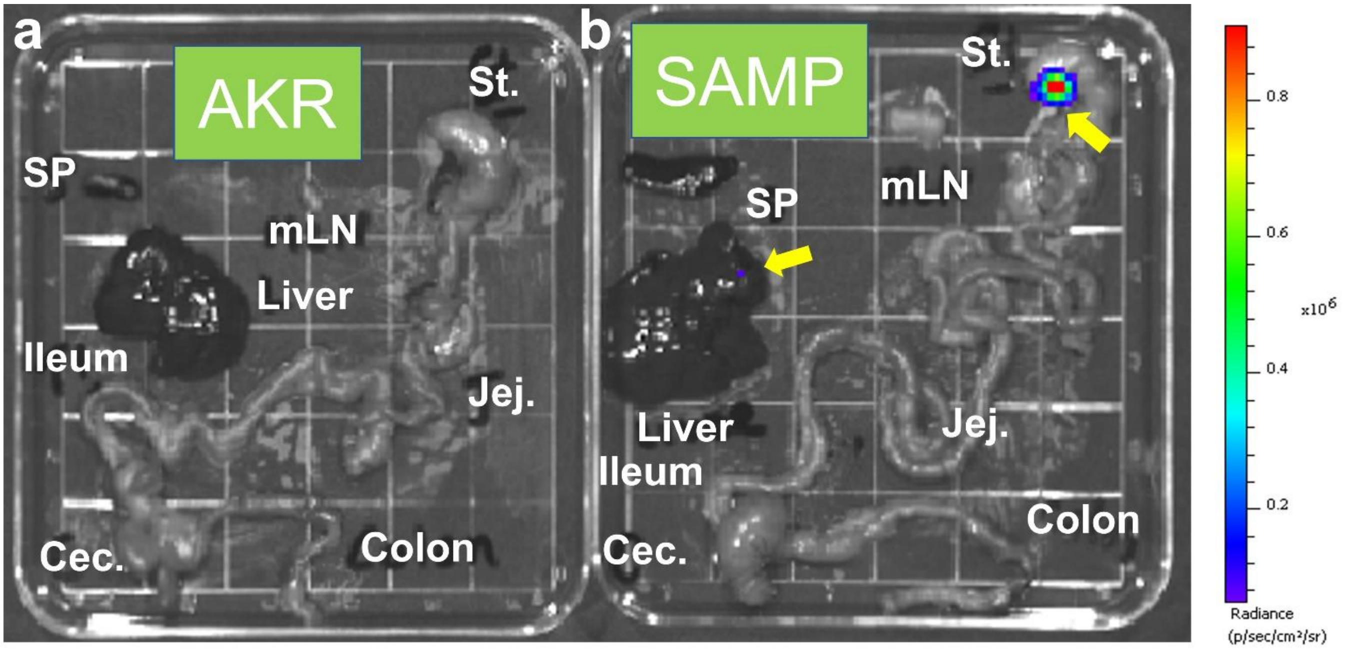

Supplementary Figure 2: Ex vivo bioluminescent imaging performed on harvested body organs showing the representative image of **(a)** AKR mice and **(b)** SAMP mice. SAMP mice show the presence of live hMSCs in the liver and the proximity of the stomach. (SP=spleen, St=Stomach, mLN=mesenteric lymph node, Jej.=Jejunum, Ileum, Cec.=Cecum). Bold arrow showing the presence of bioluminescent hMSC.

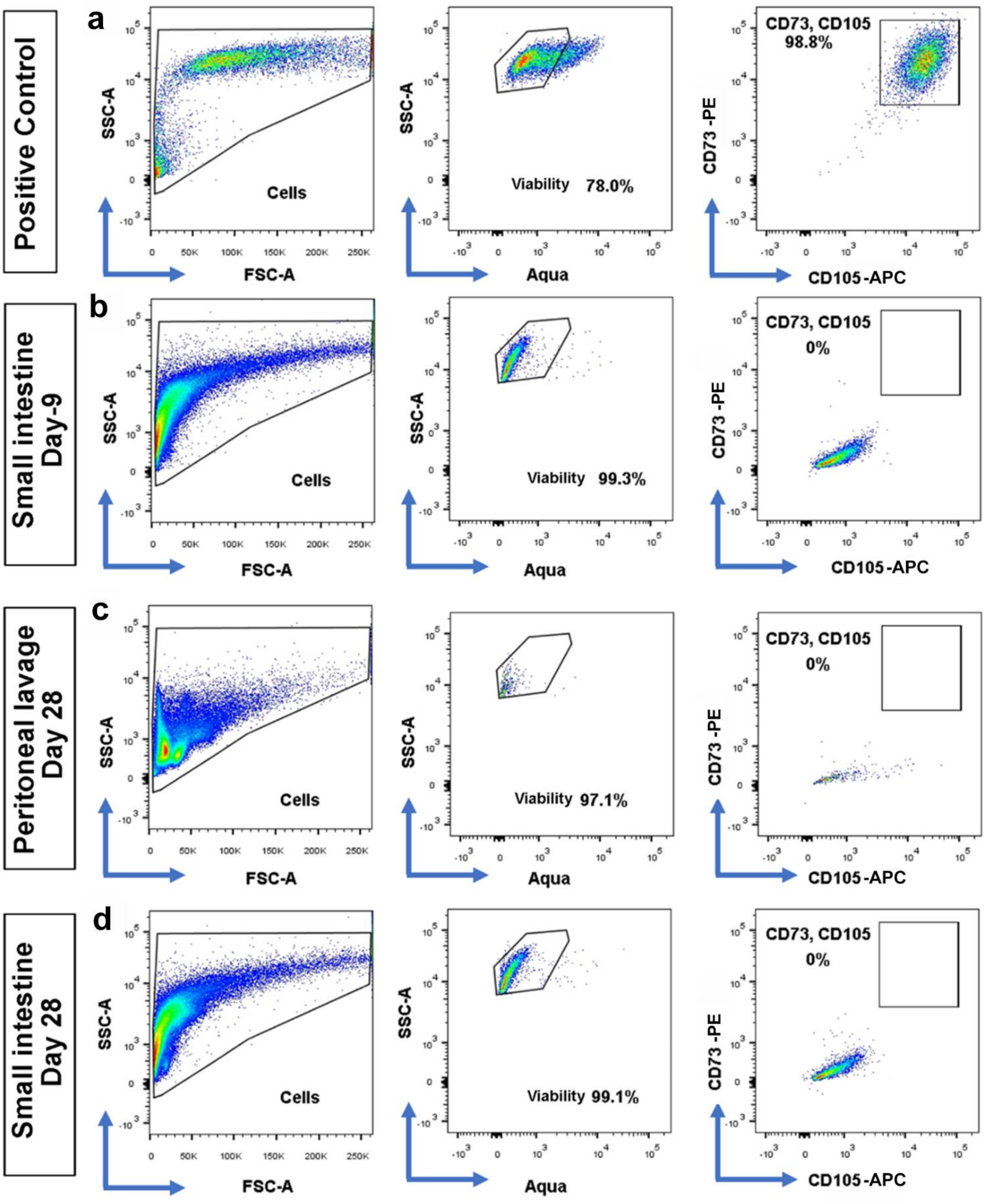

Supplementary Figure 3: Flow cytometry performed on single cell suspension from the small intestine and peritoneal lavage cell isolated at day 9 and day 28 after peritoneal administration of 5 million

IVISense680 tagged hMSC. Gating scheme showing hMSC (CD73^{+}, CD105^{+}) population. **(a)** In vitro cultured hMSC as a positive control, **(b)** Single-cell suspension of the small intestine, on day 9, **(c)** Peritoneal lavage on day 28, **(d)** Single-cell suspension of the small intestine, on day 9. Data represent 3 biological replicates.

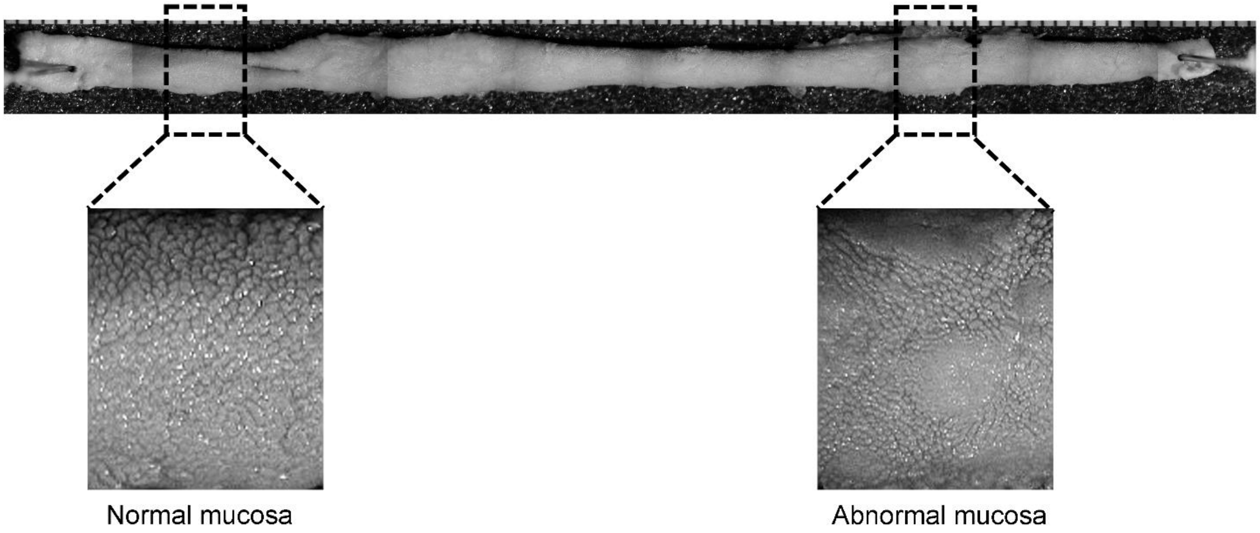

Supplementary Figure 4. Representative stereomicroscopic image of SAMP small intestine showing difference between normal and abnormal mucosa.

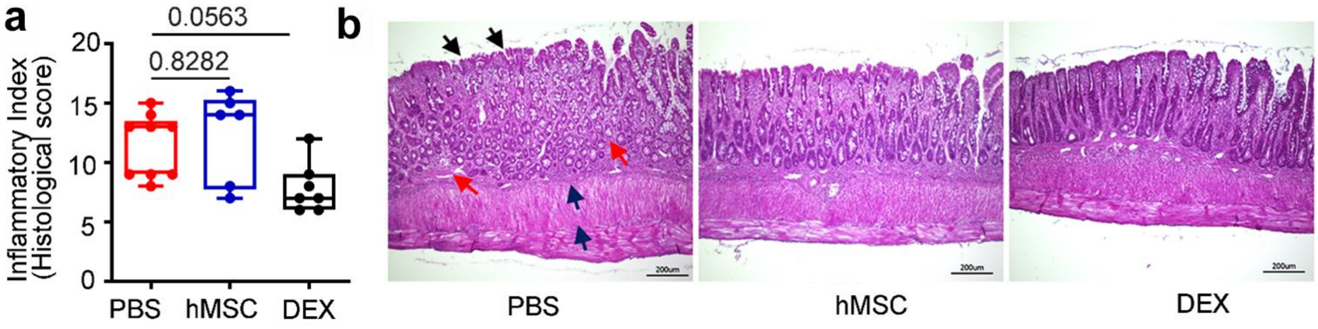

Supplementary Figure 5. SAMP mice treated with hMSC, and DEX did not achieve histological healing by day 9 (n=22 mice) **(a)** Inflammatory Index for disease severity is a histological index composed of the villus distortion index, active inflammation index, mononuclear inflammation index, chronic inflammation index and transmural inflammation index. SAMP mice treated with hMSC, and

DEX did not achieve significant histological healing (PBS:11.44±2.65 vs hMSC:12.33±3.83; P=0.8282; mean ±SD) however, a partial response is observed in DEX-treated SAMP (PBS:11.44±2.65 vs DEX:7.88±2.12; P=0.0563; mean ±SD). **(b)** Representative histopathology photomicrograph of ileum tissue from PBS, hMSC and DEX-treated groups. PBS-treated group show histological features of villus distortion (black arrow), Immune infiltrates and crypt hyperplasia (red arrow), and muscle hypertrophy (blue arrow). The scale bar represents 200µm. Data represented as box and whiskers-plot; center line: median; box limits: 25-75 percentile; whiskers: min. to max. with all data points. Data correspond to at least two independent experiments, and each data point represents one mouse.

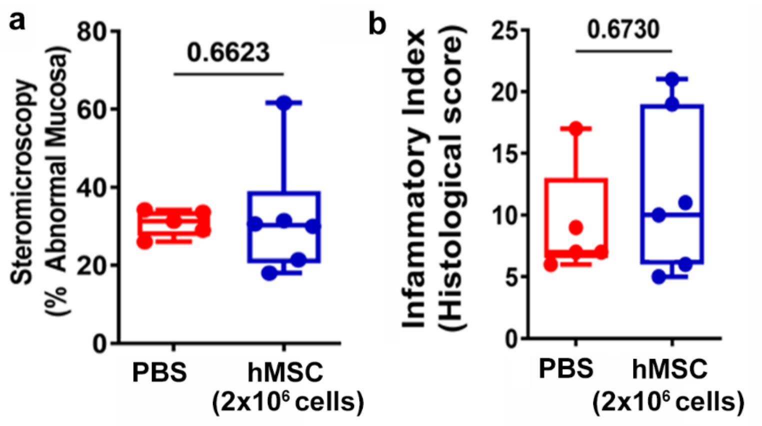

Supplementary Figure 6. SAMP mice treated with $2x10^6$ hMSC for 28 showing **(a)** Percent abnormal mucosa; no significant changes were observed in percent abnormal mucosa after $2x10^6$ hMSC treatment (PBS: 30.82±3.39 vs hMSC:32.17±15.43; P=0.6623; mean ±SD) **(b)** Inflammatory index, no significant changes were observed in inflammatory index between PBS and $2x10^6$ hMSC treated SAMP (PBS: 9.20±4.49 vs hMSC:11.29±6.34; P=0.6730; mean ±SD). Data represented as box and whiskers-plot where, center line: median; box limits: 25-75 percentile; whiskers: min. to max. with all data points. Data correspond to at least two independent experiments, and each data point represents one mouse.

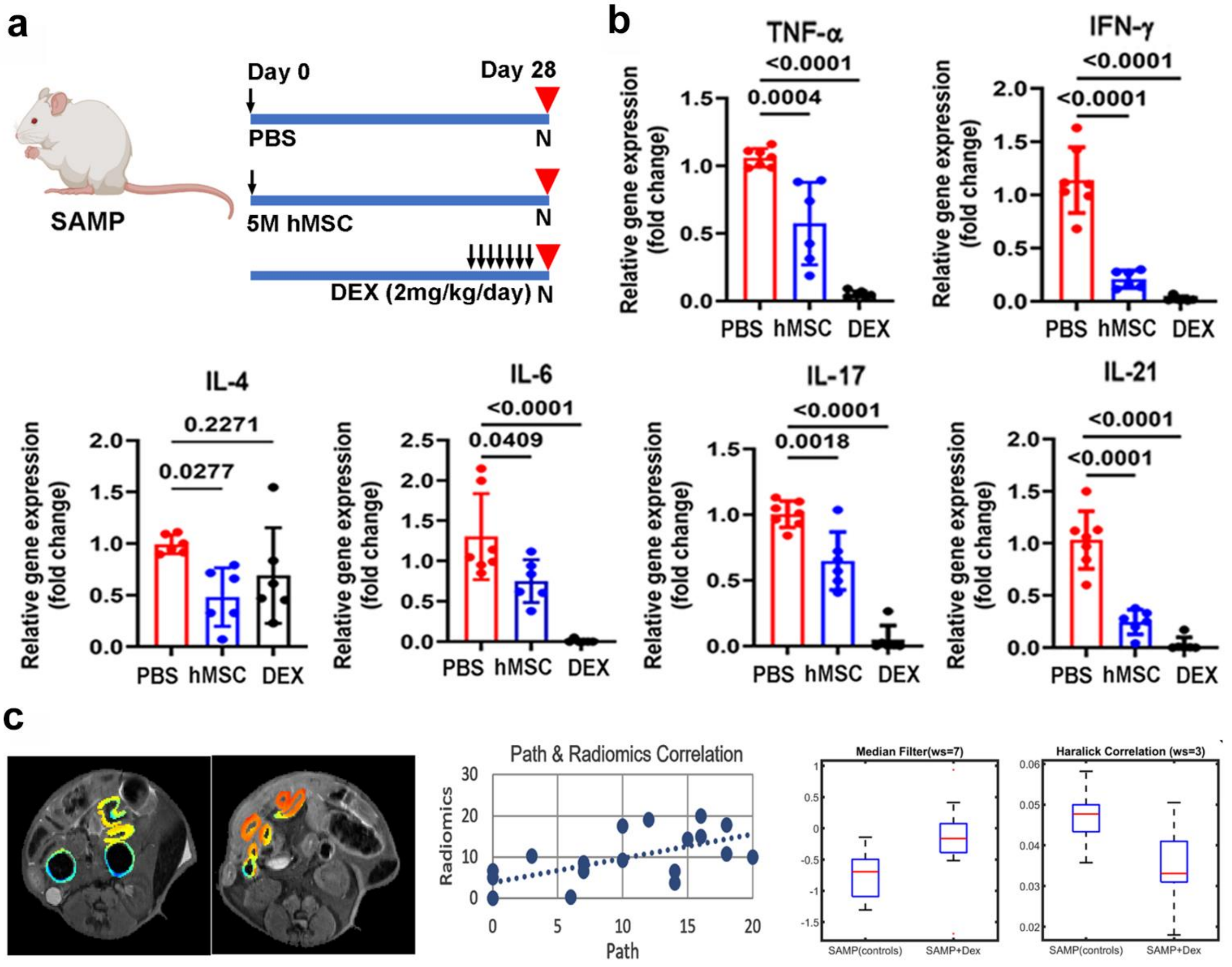

Supplementary Figure 7. (a) Schematic showing treatment regime of the experiment **(b)** Relative gene expression of Th-1, Th-2, and Th-17 pathway cytokines measured in total RNA extracted from mLN cells. The gene expression was determined by qRT-PCR, normalized to GAPDH, and expressed as fold change (2-ΔΔCt). hMSC-treated mice showed a decrease in gene expression of pro-inflammatory Th-1 cytokines TNF-α (20.57-fold change, P<0.001) and IFN-γ (0.21-fold change, P<0.0001); IL-6 (0.75-fold change, P<0.05) and Th-17 cytokine IL-17(0.65-fold change, P<0.01). DEX significantly downregulated gene expression of the majority of proinflammatory cytokines including TNF-α (0.05-fold change, P<0.0001) IFN-γ (0.02-fold change, P<0.0001), IL-6 (0.01-fold change, P<0.0001), IL-17(0.05-fold change, P<0.0001). Data represented as mean ±SD. 6-8 mice/group were studied in 3 independent experiments. **(c)** Workflow showing the statistical evaluation from MR Images; feature extraction,

correlation between path and radiomics scores, top radiomic features from SAMP mice selected via statistical testing.

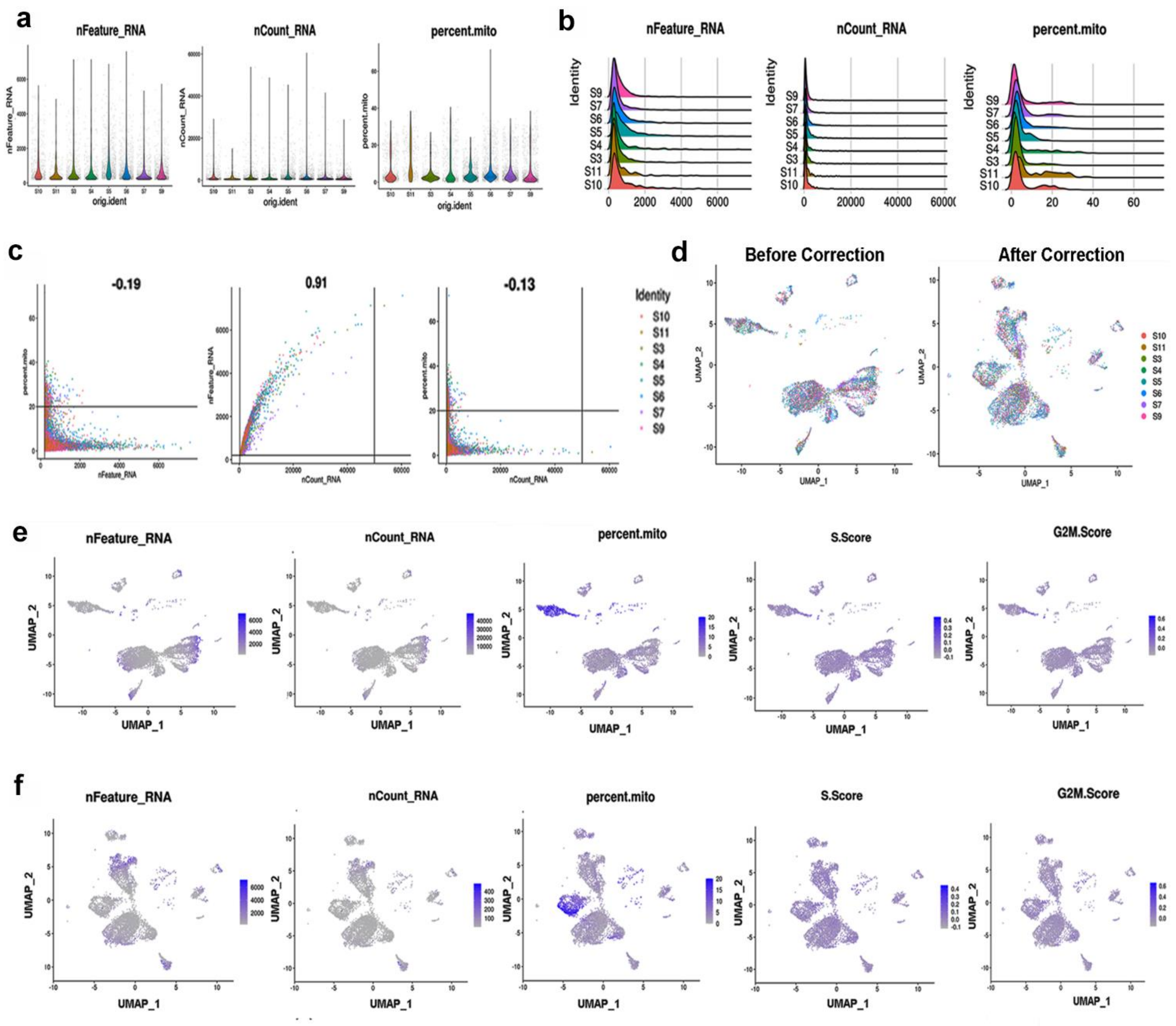

Supplementary Figure 8. Single-cell RNA sequencing data pre-processed using Seurat, version 4.1.0 running in R version 4.1.0 for quality control metrics showing **(a)**Violin plots **(b)** Ridge plots **(c)** scatter plots with proposed cutoffs **(d)** UMAP plot by sample, prior to correction and after correction **(e)** UMAP plot showing the number of UMIs/cell, number of genes/cell, percent mitochondrial gene expression, cell cycle S score, cell cycle G2M score before correction and **(f)** after correction.

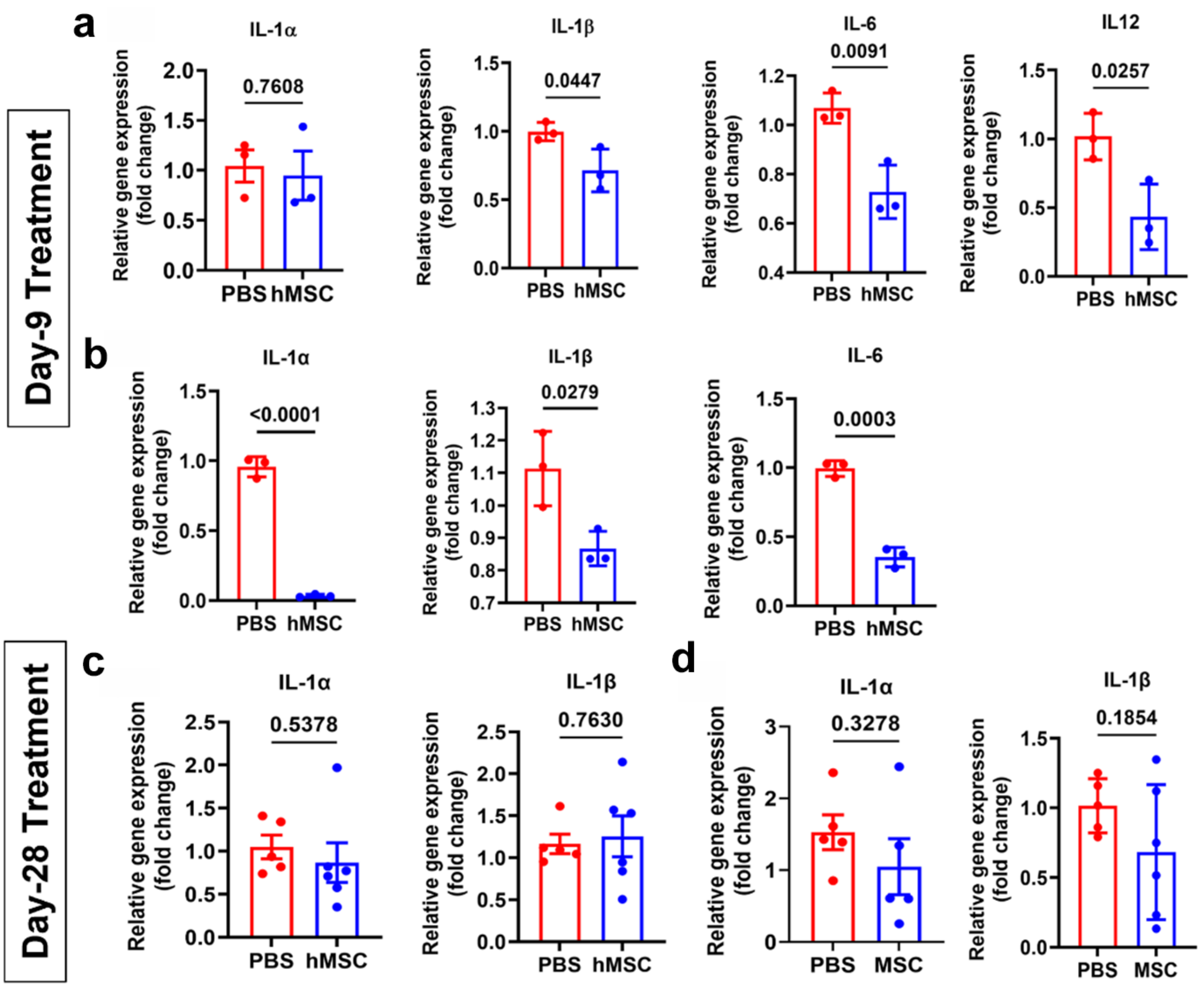

Supplementary Figure 9. Relative gene expression of cytokines measured in total RNA **(a)** hMSC treatment at day 9, resulted in the anti-inflammatory phenotype of mLN CD11b$^+$ macrophages with downregulation of IL-1β (0.71-fold change, P=0.044), IL-6 (0.71-fold change, P=0.0091) and IL-12 (0.43-fold change, P=0.025), while no change in gene expression was observed for IL-1α (P=0.76). **(b)** CD11b$^+$ macrophages isolated from SVF of mesentery also demonstrated similar anti-inflammatory phenotype with downregulation of pro-inflammatory cytokines IL-1α (0.03-fold change, P<0.0001), IL-1β (0.86-fold change, P=0.027), and IL-6 (P=0.0003). Relative gene expression of IL-1α and IL-1β did not change significantly at day 28 in **(c)** mLN and **(d)** SVF-derived CD11b$^+$ macrophages. The gene expression was determined by qRT-PCR, normalized to GAPDH, and expressed as fold change (2-ΔΔCt). Data represented as mean ±SD and correspond to at least two independent experiments; each data point represents one mouse.

Supplementary Table 1: RT-qPCR primers sequence

Gene	Forward ('5-3')	Reverse ('5-3')
GAPDH	CCCATCACCATCTTCCAGGAGC	CCAGTGAGCTTCCCGTTCAGC
TNF-α	GCCTCTTCTCATTCCTGCTTG	CTGATGAGAGGGAGGCCATT
IL-12p40	CAGAAGCTAACCATCTCCTGGTTTG	TCCGGAGTAATTTGGTG CTTCACAC
IL1-α	TCTCAGATTCACAACTGTTCGTG	AGAAAATGAGGTCGGTCTCACTA
IL1- β	CCTTCCAGGATGAGGACATGA	TGAGTCACAGAGGATGGGCTC
Arginase I	CAGAAGAATGGAAGAGTCAG	CAGATATGCAGGGAGTCACC
IL-6	GAGGATACCACTCCCAACAGACC	AGTGCATCATCGTTGTTCATACA
IFN-γ	CGACTCCTTTTCCGCTTCCTGAG	TGAACGCTACACACTGCATCTTGG
IL-4	AGATGGATGTGCCAAACGTCCTCA	AATATGCGAAGCACCTTGGAAGCC
IL-17	ATCCCTCAAAGCTCAGCGTGTC	GGGTCTTCATTGCGGTGGAGAG
IL-21	TCAGCTCCACAAGATGTAAAGGG	GGGCCACGAGGTCAATGAT
