# Supplementary material for: MSCs mediate long-term efficacy in a Crohn’s disease model by sustained anti-inflammatory macrophage programming via efferocytosis
Source: NPJ Regen Med. 2024 Jan 20;9:6. doi: 10.1038/s41536-024-00347-1 (PMC10799947; doi:10.1038/s41536-024-00347-1)
Supplement: Supplementary file 2 — Reporting summary [file 41536_2024_347_MOESM2_ESM.pdf]

Corresponding author(s): Dr Manesh dave

Last updated by author(s): Dec 15, 2023

Reporting Summary

Nature Portfolio wishes to improve the reproducibility of the work that we publish. This form provides structure for consistency and transparency in reporting. For further information on Nature Portfolio policies, see our Editorial Policies and the Editorial Policy Checklist.

Statistics

For all statistical analyses, confirm that the following items are present in the figure legend, table legend, main text, or Methods section.

n/a	Confirmed	
☐	☒	The exact sample size (*n*) for each experimental group/condition, given as a discrete number and unit of measurement
☐	☒	A statement on whether measurements were taken from distinct samples or whether the same sample was measured repeatedly
☐	☒	The statistical test(s) used AND whether they are one- or two-sided<br>*Only common tests should be described solely by name; describe more complex techniques in the Methods section.*
☐	☒	A description of all covariates tested
☐	☒	A description of any assumptions or corrections, such as tests of normality and adjustment for multiple comparisons
☐	☒	A full description of the statistical parameters including central tendency (e.g. means) or other basic estimates (e.g. regression coefficient) AND variation (e.g. standard deviation) or associated estimates of uncertainty (e.g. confidence intervals)
☐	☒	For null hypothesis testing, the test statistic (e.g. *F*, *t*, *r*) with confidence intervals, effect sizes, degrees of freedom and *P* value noted<br>*Give P values as exact values whenever suitable.*
☒	☐	For Bayesian analysis, information on the choice of priors and Markov chain Monte Carlo settings
☐	☒	For hierarchical and complex designs, identification of the appropriate level for tests and full reporting of outcomes
☒	☐	Estimates of effect sizes (e.g. Cohen's *d*, Pearson's *r*), indicating how they were calculated

Our web collection on statistics for biologists contains articles on many of the points above.

Software and code

Policy information about availability of computer code

Data collection	The details are provided in the manuscript.
Data analysis	Biorad CFX Maestro 1.0<br>GraphPad Prism Version 9.0<br>FlowJo V10.6.1<br>Amscope<br>Keyence Analysis Software (BZ-X700 Microscope)<br>Seurat, version 4.1.0<br>R version 4.1.0<br>limma, version 3.50.3<br>Living Image v.4.5<br>split-pipe v0.9.3<br>ImageJ

For manuscripts utilizing custom algorithms or software that are central to the research but not yet described in published literature, software must be made available to editors and reviewers. We strongly encourage code deposition in a community repository (e.g. GitHub). See the Nature Portfolio guidelines for submitting code & software for further information.

Data

Policy information about availability of data

All manuscripts must include a data availability statement. This statement should provide the following information, where applicable:

- Accession codes, unique identifiers, or web links for publicly available datasets
- A description of any restrictions on data availability
- For clinical datasets or third party data, please ensure that the statement adheres to our policy

Provide your data availability statement here.

Research involving human participants, their data, or biological material

Policy information about studies with human participants or human data. See also policy information about sex, gender (identity/presentation), and sexual orientation and race, ethnicity and racism.

Reporting on sex and gender	NA
Reporting on race, ethnicity, or other socially relevant groupings	NA
Population characteristics	NA
Recruitment	NA
Ethics oversight	Bone marrow was collected from healthy de-identified donors; the procedure was reviewed and approved by the University Hospitals of Cleveland and the University of California Davis Institutional Review Boards

Note that full information on the approval of the study protocol must also be provided in the manuscript.

Field-specific reporting

Please select the one below that is the best fit for your research. If you are not sure, read the appropriate sections before making your selection.

☒ Life sciences ☐ Behavioural & social sciences ☐ Ecological, evolutionary & environmental sciences

For a reference copy of the document with all sections, see nature.com/documents/nr-reporting-summary-flat.pdf

Life sciences study design

All studies must disclose on these points even when the disclosure is negative.

Sample size	No statistical method was used to determine the sample size.
Data exclusions	No data were excluded
Replication	All the experiments were repeated with 2-3 times.
Randomization	Both male and female mice with established disease (>24 weeks old) were randomly assigned to the control and treatment groups to minimize the confounders.
Blinding	All outcome assessments of the experiments including histopathology scoring, stereomicroscopy, RT qPCR, radiomics, and MRI scoring were performed in a blinded manner.

Reporting for specific materials, systems and methods

We require information from authors about some types of materials, experimental systems and methods used in many studies. Here, indicate whether each material, system or method listed is relevant to your study. If you are not sure if a list item applies to your research, read the appropriate section before selecting a response.

Materials & experimental systems

n/a	Involved in the study	
☐	☒	Antibodies
☒	☐	Eukaryotic cell lines
☒	☐	Palaeontology and archaeology
☐	☒	Animals and other organisms
☒	☐	Clinical data
☒	☐	Dual use research of concern
☒	☐	Plants

Methods

n/a	Involved in the study	
☒	☐	ChIP-seq
☐	☒	Flow cytometry
☐	☒	MRI-based neuroimaging

Antibodies

Antibodies used	APC anti-mouse CD45, clone 30-F11 BioLegend 103112<br>BV785 anti-mouse CD3, clone 17A2 BioLegend 100231<br>BV711 anti-mouse CD4, clone RM4-5 BioLegend 100549<br>BV605 anti-mouse CD8a, clone 53-6.7 BioLegend 100743<br>APC/Cy7 anti-mouse/human CD11b , clone M1/70 BioLegend 101226<br>PE/Cy-7 anti-mouse F4/80, clone BM8 BioLegend 123114<br>PE anti-human CD73, clone AD2 eBioscience 12-0739-42<br>APC anti-human CD105, clone SN6 eBioscience 17-1057-42<br>anti mouse CD16/32, clone 93 BioLegend 101302<br>Live/DeadTM fixable aqua dead cell stain kit Invitrogen L34957<br>Annexin-Pacific Blue BioLegend 640918<br>IVISense680 Fluor Cell Label Perkin Elmer NEV12001<br>CellTrackerTM Red CMTPX dye Invitrogen, C34552<br>Alexafluor 700-anti-Gr1, clone RB6-8C5 BioLegend 108421<br>APC-anti-TNFα, clone MP6-XT22 BD Pharmingen 561062<br>PE-Cy7-anti Arginase-1, clone A1exF5 Invitrogen 25-3697-82
Validation	All the antibodies were validated depending on the data provided on the manufacturer website cited literature.

Animals and other research organisms

Policy information about studies involving animals; ARRIVE guidelines recommended for reporting animal research, and Sex and Gender in Research

Laboratory animals	We used both male and female SAMP1/YitFc (SAMP) mouse with established disease (>24 weeks old).
Wild animals	NA
Reporting on sex	We used both male and female mouse in the study.
Field-collected samples	NA
Ethics oversight	Case Western Reserve University , Institutional Animal Care and Use Committee protocol # 2015-0142<br>University of California, Davis, Institutional Animal Care and Use Committee protocol protocol#21298

Note that full information on the approval of the study protocol must also be provided in the manuscript.

Plants

Seed stocks	NA
Novel plant genotypes	NA
Authentication	NA

Flow Cytometry

Plots

Confirm that:

☒ The axis labels state the marker and fluorochrome used (e.g. CD4-FITC).

☒ The axis scales are clearly visible. Include numbers along axes only for bottom left plot of group (a 'group' is an analysis of identical markers).

☒ All plots are contour plots with outliers or pseudocolor plots.

☒ A numerical value for number of cells or percentage (with statistics) is provided.

Methodology

Sample preparation	Sample preparation and methodology is described in the manuscript.
Instrument	Details are provided in the manuscript.
Software	Software details are provided in the manuscript.
Cell population abundance	Details are provided in the methods.
Gating strategy	Gating strategy is described in the figures and text.

☒ Tick this box to confirm that a figure exemplifying the gating strategy is provided in the Supplementary Information.

Magnetic resonance imaging

Experimental design

Design type	NA
Design specifications	NA
Behavioral performance measures	NA

Acquisition

Imaging type(s)	Structural MRI T2w
Field strength	7-9.4T
Sequence & imaging parameters	Scanning seq:RM, Orientation:Axial Protocol name: RARE, Echo Time(TE): 17.872-34.828, Repetition Time(TR):1542.8445-2000, Flip Angle=180,Pixel Spacing (X,Y): 0.15625-0.78125mm,Slice thickness (Z): 0.7-2mm,FOV:100x100mm,Matrix SizeX: 128-400,Matrix SizeY: 128-160,Whole Body
Area of acquisition	Whole mouse image

Diffusion MRI ☐ Used ☐ Not used

Preprocessing

Preprocessing software	NA
Normalization	NA
Normalization template	NA
Noise and artifact removal	NA
Volume censoring	NA

Statistical modeling & inference

Model type and settings	NA

Effect(s) tested | NA

Specify type of analysis: ☐ Whole brain ☒ ROI-based ☐ Both

Anatomical location(s) | *Describe how anatomical locations were determined (e.g. specify whether automated labeling algorithms or probabilistic atlases were used).*

Statistic type for inference (See Eklund et al. 2016) | Computed median, variance, Skewness, and Kurtosis of Radiomic metric within ROI

Correction | NA

Models & analysis

n/a	Involved in the study
☐	☒ Functional and/or effective connectivity
☒	☐ Graph analysis
☐	☒ Multivariate modeling or predictive analysis

Functional and/or effective connectivity | Linear Regression between generated score and pathologist ground truth

Multivariate modeling and predictive analysis | 100 radiomic features including statistics, sobel filters, GLCM,Gabor, and COLlAGE were extracted from the ROI. Wilcoxon ranksum was used to order features based in a pairwise comparison between training Mice from PBS and DEX. A Random Forest classifier trained on the training subset were then used to generate a model to predict probability of severe disease. The model was applied to all remaining groups to generate a score which was rescaled to match the range of the clinical score(0-20). This was combined with the Pathology score to generate the SIMPe score.
